# Transcriptomic and Physiological Profiling Elucidates Differential Salt Stress Responses in Tolerant ‘SO4’ and Sensitive ‘Beida’ Grapevine Rootstocks

**DOI:** 10.3390/ijms27146479

**Published:** 2026-07-21

**Authors:** Abdul Hakeem, Essam Elatafi, Wen Liu, Basma Elhendawy, Abdullah Alebidi, Rashid S. Al-Obeed, Mostafa Saeed, Jinggui Fang, Mahmoud Abdel-Sattar

**Affiliations:** 1 Key Laboratory of Genetics and Fruit Development, College of Horticulture, Nanjing Agricultural University, Nanjing 210095, China; 031424@henau.edu.cn (A.H.); eelatafi@mans.edu.eg (E.E.); 2022204028@stu.njau.edu.cn (W.L.); basmaelhendawy134@gmail.com (B.E.); 2 Department of Pomology, Faculty of Agriculture, Mansoura University, Mansoura 35516, Egypt; 3 Department of Plant Production, College of Food and Agriculture Sciences, King Saud University, P.O. Box 2460, Riyadh 11451, Saudi Arabia; aalebidi@ksu.edu.sa (A.A.); ralobeed@ksu.edu.sa (R.S.A.-O.); 4 Institute of Fruit Science, College of Agriculture and Biotechnology, Zhejiang University, Hangzhou 310058, China; mostafasaeed@zju.edu.cn

**Keywords:** abiotic stress, antioxidant enzyme, biochemical, gene expression, grape, molecular, proline, RNA-seq, rootstocks, salt stress

## Abstract

Soil salinity severely limits grapevine (*Vitis* spp.) growth and productivity, yet the mechanisms distinguishing tolerant and sensitive rootstocks remain incompletely understood. We compared the salt-tolerant rootstock ‘SO4’ with the salt-sensitive ‘Beida’ under 100 mmol L^−1^ NaCl for 0, 6, and 12 days. Salinity progressively reduced photosynthetic pigments in both genotypes, although ‘SO4’ retained higher levels. Salt treatment also increased hydrogen peroxide, malondialdehyde, soluble sugars, soluble proteins, proline, and antioxidant enzyme activities. Compared with ‘Beida’, ‘SO4’ showed stronger osmotic adjustment and greater activation of superoxide dismutase, peroxidase, catalase, and ascorbate peroxidase. RNA-seq analysis revealed extensive genotype- and time-dependent transcriptional reprogramming, with differentially expressed genes mainly associated with hormone signalling, secondary metabolism, carbon fixation, protein processing, and lipid metabolism. Weighted gene co-expression network analysis identified the MEblack module as positively associated with salt tolerance in ‘SO4’ but negatively associated with ‘Beida’. Within this module, *Vitvi01g00735/VvBCA2* and *Vitvi07g02043/VvLCB1* were prioritized as candidate hubs based on high module membership, gene significance, and intramodular connectivity. Hub-centred networks linked *VvBCA2* to redox regulation, protein homeostasis, defense, and osmotic signalling, whereas *VvLCB1* was associated with cell-wall remodelling, methyl metabolism, membrane signalling, and lipid turnover. Transcription-factor families, including MYB, WRKY, AP2/ERF, bHLH, and HSF, were more strongly represented in ‘SO4’. Collectively, these findings identify coordinated physiological and transcriptional mechanisms underlying salt tolerance and provide candidate genes for grapevine improvement.

## 1. Introduction

Salt stress is a major environmental factor that reduces plant growth and productivity worldwide, especially in arid and semi-arid areas [1]. Salt-affected soils are a major problem worldwide. The Food and Agriculture Organization of the United Nations reports that over 833 million hectares are affected, representing 8.7% of the planet’s land area [2]. Other reports say that this number could be as high as 1.4 billion hectares, or 10.7% of all land [3]. The problem is worse in naturally dry and semi-dry areas of Africa, Asia, and Latin America, which have more than two-thirds of the world’s salt-affected soils [4]. Furthermore, an estimated 20% to 50% of irrigated land globally is salt-affected, posing a major threat to food production systems [2].

High soil salinity has two main negative effects, osmotic stress and ion toxicity [5], where osmotic stress arises from reduced water potential that induces physiological drought [6,7,8], and ion toxicity results from the accumulation of ions such as sodium (Na^+^) and chloride (Cl^−^), which disrupts metabolism and impairs the uptake of nutrients [7,9]. These main pressures have secondary consequences, such as oxidative stress, which occurs when excessive reactive oxygen species (ROS) are generated and damage cellular structures [6,10]. Because of this, salt-stressed plants show several adverse effects, including impaired seed germination, stunted growth, chlorosis, and premature senescence [9,11]. Stomatal closure, lower chlorophyll concentration, and damage to the photosynthetic machinery all make photosynthesis significantly impaired [9,12]. In woody perennial crops like grapevines, these physiological disruptions severely restrict vegetative growth and diminish long-term vineyard productivity [13].

Grapes (*Vitis vinifera* L.) are an economically vital fruit crop cultivated globally for wine, juice, raisins, and fresh consumption and are widely recognized for their nutritional and health-promoting properties [14,15,16,17]. China is the world’s largest grape producer, accounting for about 15% to 17% of global production. In 2023, China produced more than 13.57 million tons of grapes [18]. The country’s many climates and modern farming methods have made it possible for grape cultivation to expand rapidly, especially in places like Xinjiang, Shandong, and Yunnan [19]. China’s position in the global grape industry has strengthened even further as grape quality and yields have improved.

Although grapevines have the potential to adapt, they are increasingly susceptible to numerous environmental stresses [14,20,21,22,23]. Soil salinization is becoming a significant threat to grape cultivation worldwide. Salt stress negatively impacts grapevines by reducing water availability due to osmotic stress, increasing the toxicity of sodium (Na^+^) and chloride (Cl^−^) ions, and hindering plant development, fruit yield, and quality [13]. Furthermore, exacerbated by climate change and poor irrigation practices, salinity impairs photosynthetic activity, ion homeostasis, chlorophyll metabolism, flavonoid integration, transpiration, cellular signaling, osmotic balance, and water use efficiency [24,25], which could make it impossible to cultivate grapes in some areas. Thus, researchers are currently working to identify grapevine rootstocks that can tolerate salt and to develop cultivation methods that mitigate these negative effects [13]. This will ensure that grapevines can continue to produce and thrive in saline conditions.

Using grapevine rootstocks is a common practice in modern viticulture to mitigate the effects of abiotic stress, especially excessive salinity [26,27]. Grafting commercial *Vitis vinifera* scions onto well-chosen rootstocks can make them far more resistant to adverse weather conditions, such as drought stress, salt stress, heavy metals, and iron chlorosis [26,27]. When salt stress occurs, rootstocks employ various physiological and molecular mechanisms to protect the grapevine. Tolerant rootstocks can prevent toxic ions such as Na^+^ and Cl^−^ from moving from the soil to the shoots and leaves. This process, known as ion exclusion, is a major physiological determinant of salt tolerance in grapevines, protecting the photosynthetic machinery in the scion from toxic accumulation [28,29]. These rootstocks usually have a higher potassium-to-Na^+^ ratio, which is important for cellular function [30]. In addition, tolerant rootstocks can trigger several defence responses when exposed to salt, such as activating genes that regulate stress signalling and hormone homeostasis and producing defensive chemicals, such as proline and antioxidant enzymes [13,29]. This genetic and physiological flexibility helps the grapevine cope with osmotic stress and protect its cells from harm. Such flexibility allows the grapevine to keep growing and photosynthesizing even under saline conditions [29,31]. So, choosing rootstocks that have been shown to tolerate salt is a crucial way to keep grape production going in areas where the soil is becoming saline [32].

Recent transcriptomic studies in grapevine have elucidated specific molecular mechanisms underlying salt stress responses. For instance, salt-tolerant rootstocks exhibit restricted Na^+^ transport via the regulation of *VvSOS1* and *VvNHX* antiporters, maintaining ion homeostasis [5]. Physiologically, this is accompanied by the rapid accumulation of osmolytes and the upregulation of antioxidant genes, such as *VvSOD* and *VvCAT*, to mitigate ROS bursts [33]. At the transcriptional level, specific TF families, including WRKY, MYB, and ERF, have been identified as central regulators that modulate these downstream defense pathways in response to salinity [34,35]. Despite these advances, we still do not know much about how grapevine plants, especially the ‘SO4’ and ‘Beida’ rootstocks, coordinate these physiological and gene expression levels to differentiate tolerance from sensitivity.

Therefore, the primary objective of our study was to uncover the underlying physiological and molecular mechanisms that differentiate salt-tolerant from salt-sensitive grapevine rootstocks. To accomplish these objectives, we concentrated on two divergent genotypes: ‘SO4’ (salt-tolerant) and ‘Beida’ (salt-sensitive). We investigated their phenotypic traits, physicochemical responses, and gene expression changes under 100 mmol L^−1^ salt stress over a period of 0, 6, and 12 days. We also used transcriptomic data to look at how gene expression differed between the two rootstocks and found important salinity-responsive transcription factors (TFs) and co-expression modules. By directly comparing a tolerant and a sensitive rootstock, our study highlights the crucial genes and pathways governing salt tolerance, providing a valuable theoretical foundation for breeding salt-resistant grapevine varieties. We hypothesized that the superior salt tolerance of ‘SO4’ is driven by a more robust and coordinated activation of osmotic adjustment, antioxidant defense, and specific transcription factor networks compared to ‘Beida’.

## 2. Results

### 2.1. Physiological and Biochemical Responses to Salt Stress

**Figure 1:**
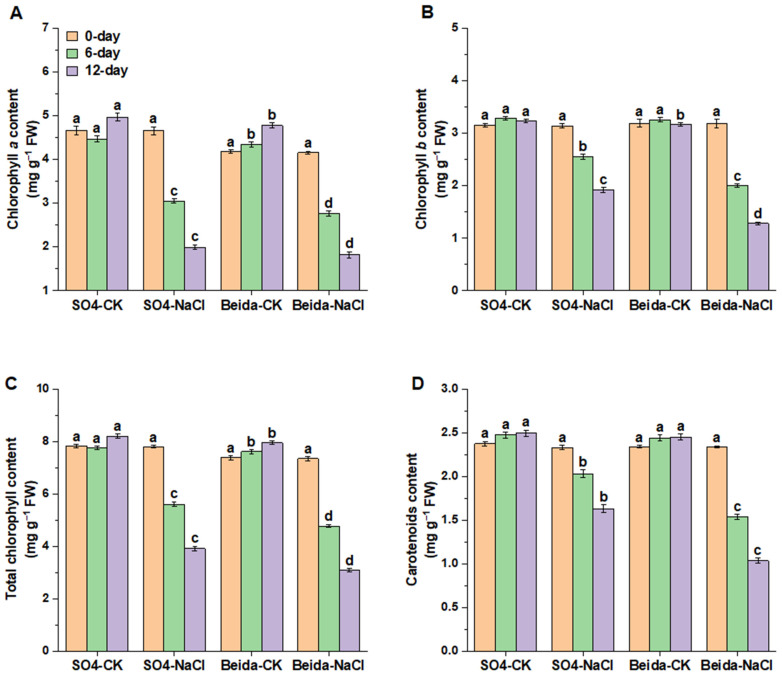
Impact of salt stress (NaCl; 100 mmol L^−1^) on the contents of chlorophyll *a* (**A**), chlorophyll *b* (**B**), total chlorophyll (**C**), and carotenoids (**D**) of two grapevine rootstocks (SO4 and Beida) at 0, 6, and 12 days of stress. The value denotes the mean ± standard deviation derived from independent biological replicates. Two factors, salt concentrations and rootstocks, were analyzed using a two-way analysis of variance with three replicates. Letters assigned to vertical bars indicate significant differences, as determined by Tukey’s HSD test at *p* < 0.05.

To understand the physiological impact of salt stress (100 mmol L^−1^ NaCl) on grapevine rootstocks over a 12-day period, we evaluated changes in photosynthetic pigments, osmolytes, ROS, and antioxidant enzyme activities. Under salt stress, both ‘SO4’ and ‘Beida’ rootstocks exhibited a progressive decline in chlorophyll a, chlorophyll b, total chlorophyll, and carotenoid contents compared to their respective controls (Figure 1A–D). However, the ‘SO4’ rootstock demonstrated a superior ability to maintain these photosynthetic pigments under stress compared to ‘Beida’.

**Figure 2:**
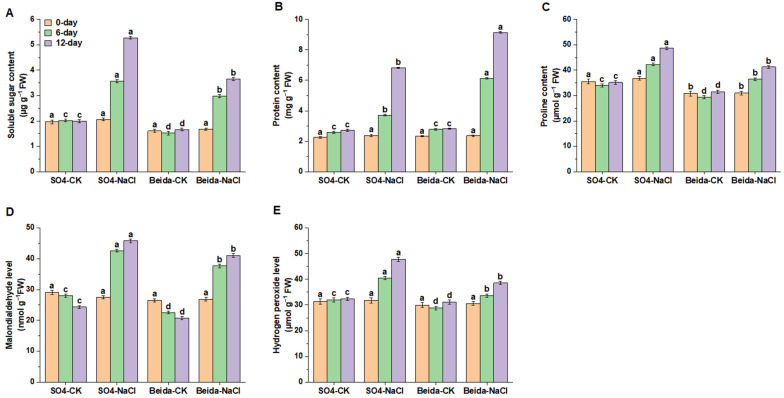
Impact of salt stress (NaCl; 100 mmol L^−1^) on the contents of soluble sugar (**A**), protein (**B**), proline (**C**), malondialdehyde (**D**), and hydrogen peroxide (**E**) of two grapevine rootstocks (SO4 and Beida) at 0, 6, and 12 days of stress. The value denotes the mean ± standard deviation derived from independent biological replicates. Two factors, salt concentrations and rootstocks, were analyzed using a two-way analysis of variance with three replicates. Letters assigned to vertical bars indicate significant differences as determined by Tukey’s HSD test at *p* < 0.05.

In response to osmotic and oxidative challenges induced by salinity, the rootstocks altered their biochemical profiles significantly. Soluble sugars and proline (critical osmoprotectants) increased markedly by day 12, with ‘SO4’ accumulating the highest concentrations of both compounds (Figure 2A,C). Conversely, total protein content was highest in the susceptible ‘Beida’ rootstock (Figure 2B). As expected during salinity stress, lipid peroxidation (measured via malondialdehyde; MDA) and H_2_O_2_ concentrations rose significantly on days 6 and 12 (Figure 2D,E). Interestingly, ‘SO4’ exhibited the highest accumulation of H_2_O_2_ and MDA.

**Figure 3:**
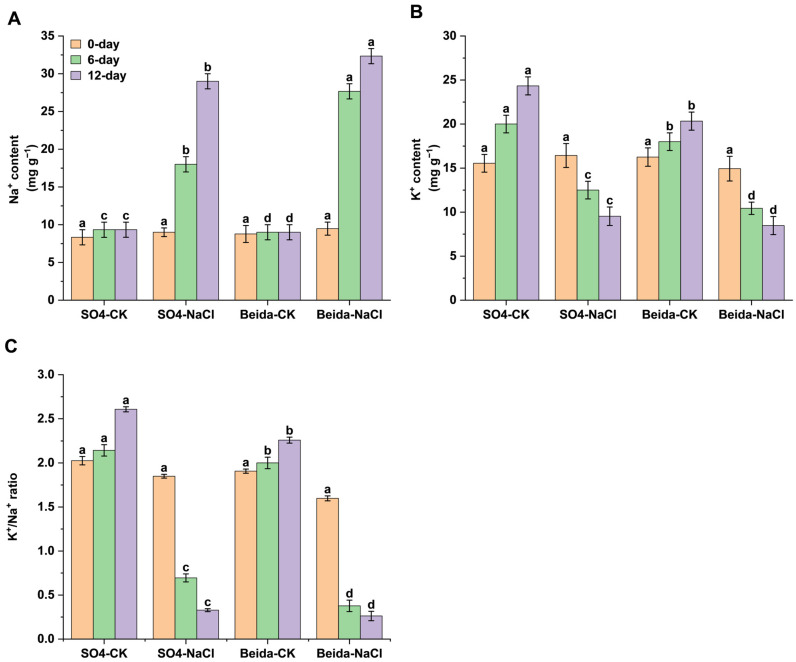
Effects of salt (NaCl; 100 mmol L^−1^) stress on ion accumulation and homeostasis in the leaves of ‘SO4’ and ‘Beida’ grapevine rootstocks. (**A**) sodium (Na^+^) content; (**B**) potassium (K^+^) content; (**C**) K^+^/Na^+^ ratio. Measurements were taken at 0, 6, and 12 days (d) under control (CK) and NaCl treatment conditions. Data are presented as the mean ± standard error (SE) of three biological replicates. Different lowercase letters above the bars indicate statistically significant differences among all groups according to a two-way ANOVA followed by Tukey’s HSD test at *p* < 0.05.

To evaluate the ionic homeostasis of ‘SO4’ and ‘Beida’ rootstocks under salt stress, the Na^+^ content, K^+^ content, and K^+^/Na^+^ ratio were measured at 0, 6, and 12 days of treatment (Figure 3). At 0 day, no significant differences in Na^+^ content were observed among any of the groups (Figure 3A). However, following NaCl treatment, Na^+^ accumulation increased significantly in both rootstocks in a time-dependent manner. Notably, at 6 days of salt stress, the Na^+^ content in ‘Beida’ (27.67 mg g^−1^) was significantly higher than that in ‘SO4’ (18.00 mg g^−1^). By 12 days, both rootstocks reached their peak Na^+^ accumulation, with ‘Beida’ (32.33 mg g^−1^) remaining significantly higher than ‘SO4’ (29.00 mg g^−1^), while the control groups maintained stable, low Na^+^ levels. Conversely, K^+^ content exhibited an opposite trend (Figure 3B). Under control conditions, K^+^ levels remained relatively stable across all time points. Upon exposure to NaCl, K^+^ content progressively decreased. Importantly, ‘SO4’ maintained a significantly higher K^+^ content compared to ‘Beida’ at both 6 days and 12 days. Reflecting the contrasting dynamics of Na^+^ accumulation and K^+^ depletion, the K^+^/Na^+^ ratio dropped sharply under salt stress (Figure 3C). While control plants maintained a balanced ratio, salt-stressed plants showed a severe imbalance. ‘SO4’ maintained a significantly higher K^+^/Na^+^ ratio compared to ‘Beida’ at both 6 days and 12 days. These results indicate that the ‘SO4’ rootstock possesses a superior capacity to restrict Na^+^ influx and retain K^+^, thereby maintaining a more favorable ionic balance under prolonged salt stress compared to ‘Beida’.

**Figure 4:**
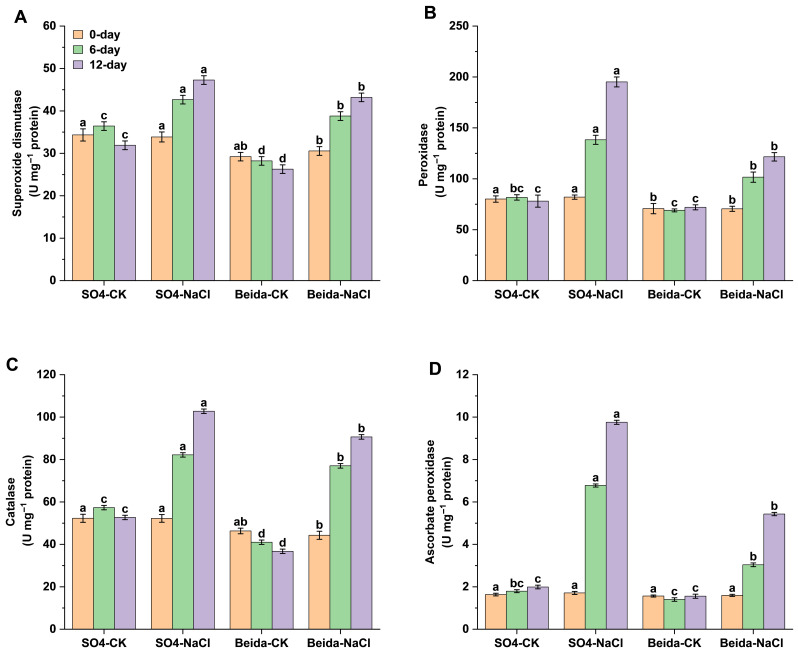
Impact of salt stress (NaCl; 100 mmol L^−1^) on the levels of superoxidase dismutase (**A**), peroxidase (**B**), catalase (**C**), and ascorbate peroxidase (**D**) of two grapevine rootstocks (SO4 and Beida) at 0, 6, and 12 days of stress. The value denotes the mean ± standard deviation derived from independent biological replicates. Two factors, salt concentrations and rootstocks, were analyzed using a two-way analysis of variance with three replicates. Letters assigned to vertical bars indicate significant differences as determined by Tukey’s HSD test at *p* < 0.05.

To counteract this severe oxidative burst, the rootstocks activated their enzymatic antioxidant defense systems. Salt stress markedly enhanced the activities of SOD, POD, CAT, and APX. Consistent with its higher ROS levels and greater overall tolerance, the ‘SO4’ rootstock induced significantly higher activities of all four antioxidant enzymes compared to ‘Beida’ and the control groups, particularly by day 12 (Figure 4A–D).

### 2.2. Transcriptome Reprogramming Aligns with Physiological Stress Responses

**Figure 5:**
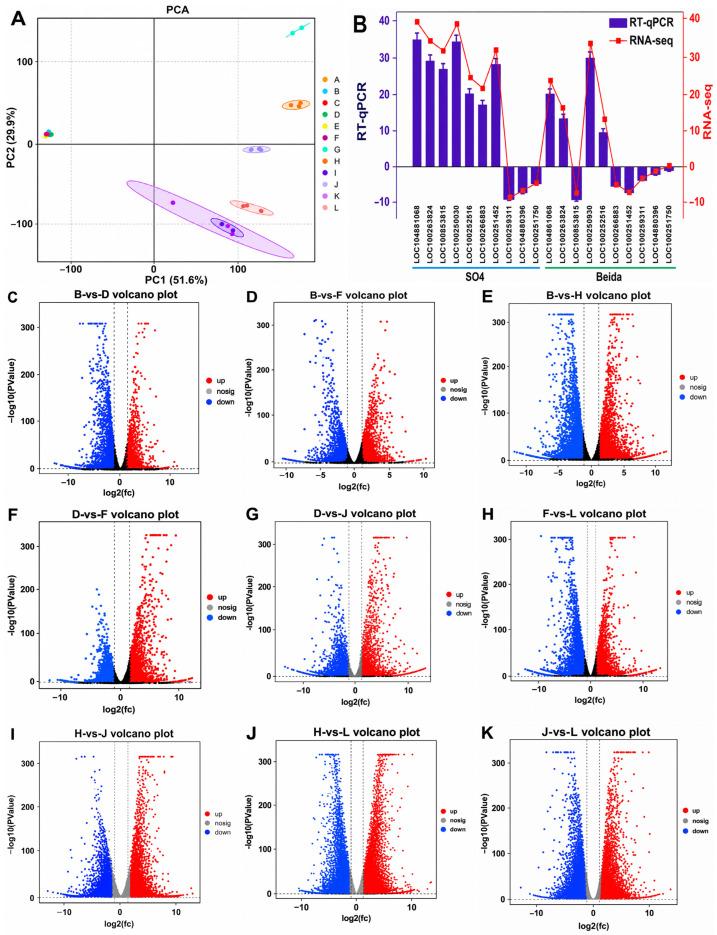
Transcriptional profiling of ‘SO4’ and ‘Beida’ grapevine rootstocks under salt stress. (**A**) Principal component analysis (PCA) of gene expression patterns across all samples. (**B**) Validation of RNA-seq data by RT-qPCR analysis following 12 days of salt stress treatment. The violet bars represent the relative expression levels obtained from RT-qPCR, while the red bars indicate the FPKM values derived from RNA-seq analysis. (**C**–**K**) Volcano plots illustrating the differentially expressed genes (DEGs) between comparison groups of ‘SO4’ and ‘Beida’ grapevine rootstocks following 0, 6, and 12 days of NaCl treatment. The *x*-axis represents the log_2_-fold change (FC) values, and the *y*-axis represents −log**_10_**(*p*-value). Red dots signify upregulated DEGs, while blue dots denote downregulated DEGs. The comparison groups are as follows: B vs. D (0 d vs. 6 d of NaCl) (**C**), B vs. F (0 d vs. 12 d of NaCl) (**D**), B vs. H (0 d vs. 0 d of NaCl) (**E**), D vs. F (6 d vs. 12 d) (**F**), D vs. J (6 d vs. 6 d of NaCl) (**G**), J vs. L (6 d vs. 12 d of NaCl) (**H**), F vs. L (12 d vs. 12 d of NaCl) (**I**), H vs. J (0 d vs. 6 d of NaCl) (**J**), and H vs. L (0 d vs. 12 d of NaCl) (**K**).

To investigate the genetic mechanisms driving the observed physiological differences, we conducted comprehensive RNA-seq analysis on 36 samples from both rootstocks. Sequencing yielded an average of over 128 billion clean bases per biological replicate, with highly reliable Q2 and Q3 quality scores (>97% and >93%, respectively; Supplementary Table S2). Principal component analysis (PCA) revealed clear distinct clustering between the treated and non-treated groups, as well as distinct temporal separation between 6 and 12 days of stress (Figure 5A). Additionally, to validate the RNA-seq data, we selected several DEGs that exhibited high fold-changes and were functionally relevant to salt stress (such as key TFs and antioxidant-related genes) for RT-qPCR analysis (Figure 5B). RNA-seq reliability was successfully validated via RT-qPCR. Differential expression analysis highlighted a massive transcriptional response to salinity. Volcano plot analyses across nine pairwise comparisons demonstrated that the majority of DEGs were significantly up-regulated following salt treatment (Figure 5C–K).

**Figure 6:**
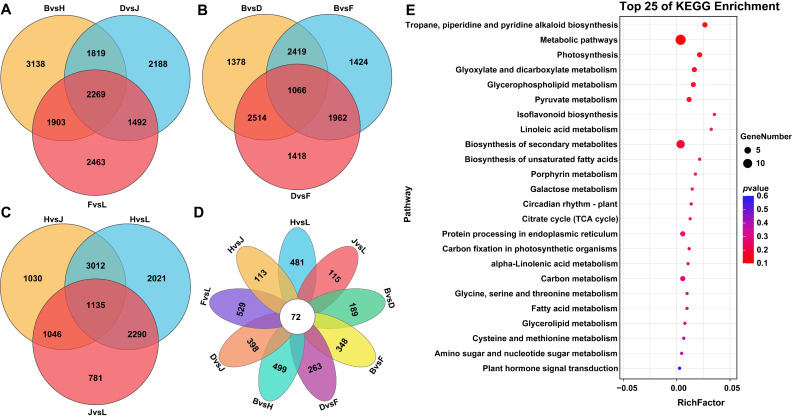
Multivariate annotation of differentially expressed genes in the ‘SO4’ and ‘Beida’ grapevine rootstocks in response to salt stress. (**A**–**D**) Venn diagrams representing the overlapping and unique differentially expressed genes (DEGs) between specific comparison groups of ‘SO4’ and ‘Beida’ grapevine rootstocks under salt stress treatments. The numbers within each region indicate the count of DEGs shared or unique to the respective comparisons. (**E**) Top 25 KEGG enrichment analysis of DEGs identified between ‘SO4’ and ‘Beida’ grapevine rootstocks. The scatter plot displays enriched pathways on the *y*-axis, with RichFactor on the *x*-axis. Dot size corresponds to the number of genes (GeneNumber) associated with each pathway, and dot color represents the *p*-value significance level (www.kegg.jp/kegg/kegg1.html; accessed on 3 March 2024).

Gene Ontology (GO) enrichment analysis of DEGs from Profile 0 to Profile 5 indicated that the majority of annotated genes were positively classified into biological processes, cellular components, and molecular functions in grapevine rootstocks subjected to salt stress (Supplementary Figure S3). Crucially, GO and KEGG enrichment analyses directly mirrored our physiological observations. Reflecting the significant degradation of leaf pigments (Figure 1), DEGs were highly enriched in pathways related to “photosynthesis,” “chloroplast stroma,” and “porphyrin metabolism.” Furthermore, aligning directly with the massive accumulation of H_2_O_2_ and the subsequent spike in antioxidant enzyme activities (Figure 3 and Figure 4), GO terms such as “response to hydrogen peroxide” and “response to a toxic substance” were significantly enriched (Supplementary Figure S1G–I). Broadly, the KEGG pathway analysis indicated that genotype-specific DEGs were heavily concentrated in metabolic pathways, such as glyoxylate and dicarboxylate metabolism, glycerophospholipid metabolism, and the biosynthesis of secondary metabolites (e.g., isoflavonoids and unsaturated fatty acids), as well as in plant hormone signal transduction and endoplasmic reticulum protein processing (Figure 6E, Supplementary Figure S2). Venn diagrams (Figure 6A–C) illustrated the overlapping and unique DEGs across the different treatment time points and genotypes, highlighting that ‘SO4’ possessed a larger pool of unique stress-responsive transcripts compared to ‘Beida’. Additionally, Figure 6D provided a comprehensive visual representation of all DEGs across samples, with the petals specifically displaying genes uniquely expressed at various time points in both rootstocks.

### 2.3. Co-Expression Network Analysis Highlights Key Salt-Tolerance Pathways

**Figure 7:**
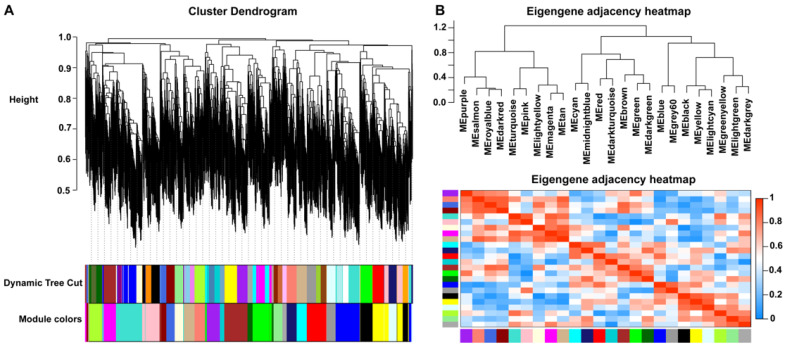
Weighted gene co-expression network analysis (WGCNA) analysis of salt stress responses of DEGs in ‘SO4’ and ‘Beida’ grapevine rootstocks. (**A**) Gene cluster dendrograms and module colors. (**B**) Illustration of Eigengene adjacency heatmap summarizes the gene expression profile of each module. The red color is presented as upregulated, and the blue color is presented as downregulated.

To identify specific gene networks regulating salt tolerance, we utilized WGCNA. WGCNA clustered the DEGs into 24 distinct expression modules (Figure 7A,B; Supplementary Tables S3 and S4, Supplementary Figure S4). The ‘MEblack’ module (containing 274 DEGs) was identified as the most positively correlated regulatory network in the tolerant ‘SO4’ rootstock, while showing a negative correlation in ‘Beida’ under salt stress. Furthermore, module eigengene analysis revealed that MEblack was consistently upregulated in ‘SO4’ rootstock under salt stress, reaching its highest expression at 12 days of NaCl treatment (ME = 0.346), while it was progressively downregulated in ‘Beida’ under the same conditions, reaching its lowest level at 12 days (ME = −0.255) (Supplementary Table S3). This divergent pattern indicates that the 111 genes comprising this module are specifically activated in the tolerant genotype and repressed in the sensitive one, suggesting a collective protective role under salt stress. The gene cluster dendrogram (Figure 7A) illustrates the hierarchical clustering of gene expression dissimilarities, which were then assigned to distinct color-coded modules. The eigengene adjacency heatmap (Figure 7B) summarizes the topological overlap relationships among these modules, demonstrating that distinct co-expression networks are independently regulated in response to salt stress. Furthermore, intramodular connectivity analysis within the MEblack module identified specific hub genes (detailed in Supplementary Table S5). Intramodular connectivity analysis identified 10 hub genes within the MEblack module (kME > 0.94, GS > 0.81), with *Vitvi01g00735* exhibiting the highest overall connectivity (Degree = 848.4).

### 2.4. Hub-Centred Co-Expression Networks Identify Candidate Salinity-Responsive Genes

**Figure 8:**
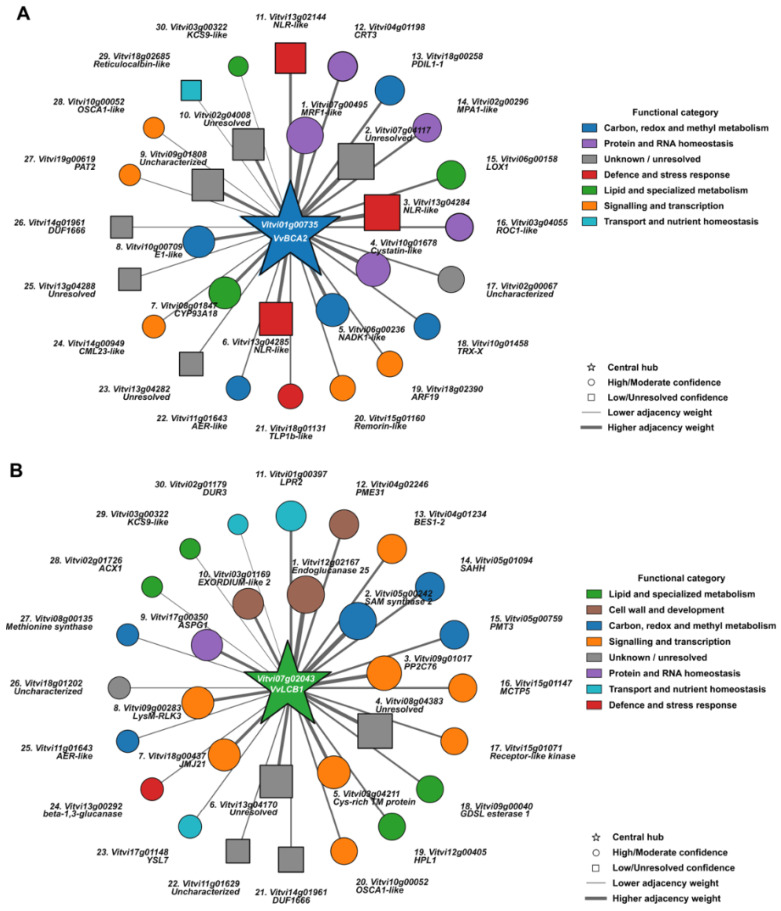
Hub-centred co-expression networks of candidate salinity-associated genes in the MEblack module. (**A**) Network centred on *Vitvi01g00735/VvBCA2* and its 30 strongest directly connected genes, ranked by WGCNA soft-thresholded adjacency weight. The network comprised 31 nodes and 30 edges, with adjacency weights ranging from 0.1776 to 0.2792. (**B**) Network centred on *Vitvi07g02043/VvLCB1* and its 30 strongest directly connected genes, with adjacency weights ranging from 0.2637 to 0.3892. Central hub genes are represented by enlarged stars, while neighbouring genes are displayed as circles or squares. Node colours indicate broad functional categories, circles represent genes with high or moderate annotation confidence, and squares represent genes with low-confidence or unresolved annotations. Edge width and neighbouring-node size are proportional to the soft-thresholded adjacency weight connecting each gene to the central hub.

The *VvBCA2*-centred network comprised 31 nodes and 30 edges, with adjacency weights ranging from 0.1776 to 0.2792 (Figure 8A; Supplementary Tables S6 and S7). The strongest connections involved *Vitvi07g00495/MRF1-like*, *Vitvi07g04117*, *Vitvi13g04284/NLR-like*, *Vitvi10g01678/Cystatin-like*, and *Vitvi06g00236/NADK1-like*. The network also contained genes associated with redox regulation (*TRX-X*), protein folding and quality control (*CRT3*, *PDIL1-1*, and *ROC1-like*), oxylipin metabolism (*LOX1*), auxin signalling (*ARF19*), calcium and osmotic signalling (*CML23-like and OSCA1-like*), reactive-carbonyl detoxification (*AER-like*), and very-long-chain fatty acid synthesis (*KCS9-like*). Furthermore, the *VvLCB1*-centred network also contained 31 nodes and 30 edges, with adjacency weights ranging from 0.2637 to 0.3892 (Figure 8B; Supplementary Tables S8 and S9). The genes with the highest adjacency weights were *Vitvi12g02167/Endoglucanase 25*, *Vitvi05g00242/SAM synthase 2*, *Vitvi09g01017/PP2C76*, *Vitvi08g04383*, and *Vitvi03g04211/Cys-rich TM protein*. This network contained genes associated with cell-wall remodelling, methionine and S-adenosylmethionine metabolism, receptor and protein phosphatase signalling, chromatin regulation, osmotic calcium signalling, oxylipin metabolism, fatty-acid turnover, and nutrient transport. However, several unresolved or poorly characterized genes were prioritized through their strong connections with the selected central genes. These included *Vitvi07g04117*, *Vitvi02g04008*, *Vitvi13g04282*, and *Vitvi13g04288* in the *VvBCA2* network, and *Vitvi08g04383*, *Vitvi13g04170*, *Vitvi11g01629*, and *Vitvi18g01202* in the *VvLCB1* network. Four genes, *OSCA1-like*, *AER-like*, *KCS9-like*, and *DUF1666*, were shared between the two top-30 networks, indicating potential points of convergence between the two co-expression neighbourhoods.

### 2.5. Dynamic Expression Profiles Reveal Metabolic Reprogramming Associated with Salt Tolerance

**Figure 9:**
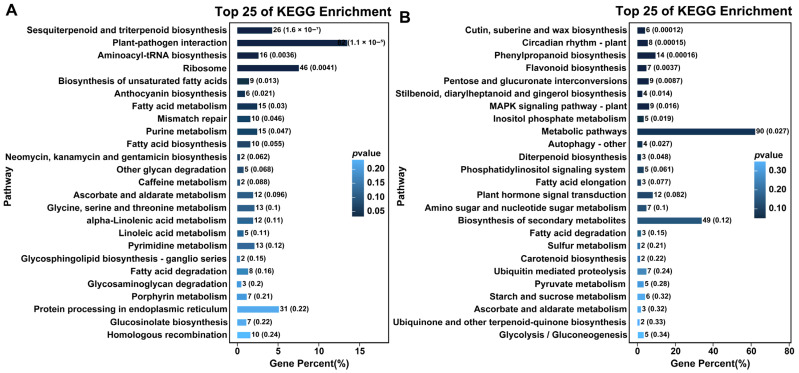

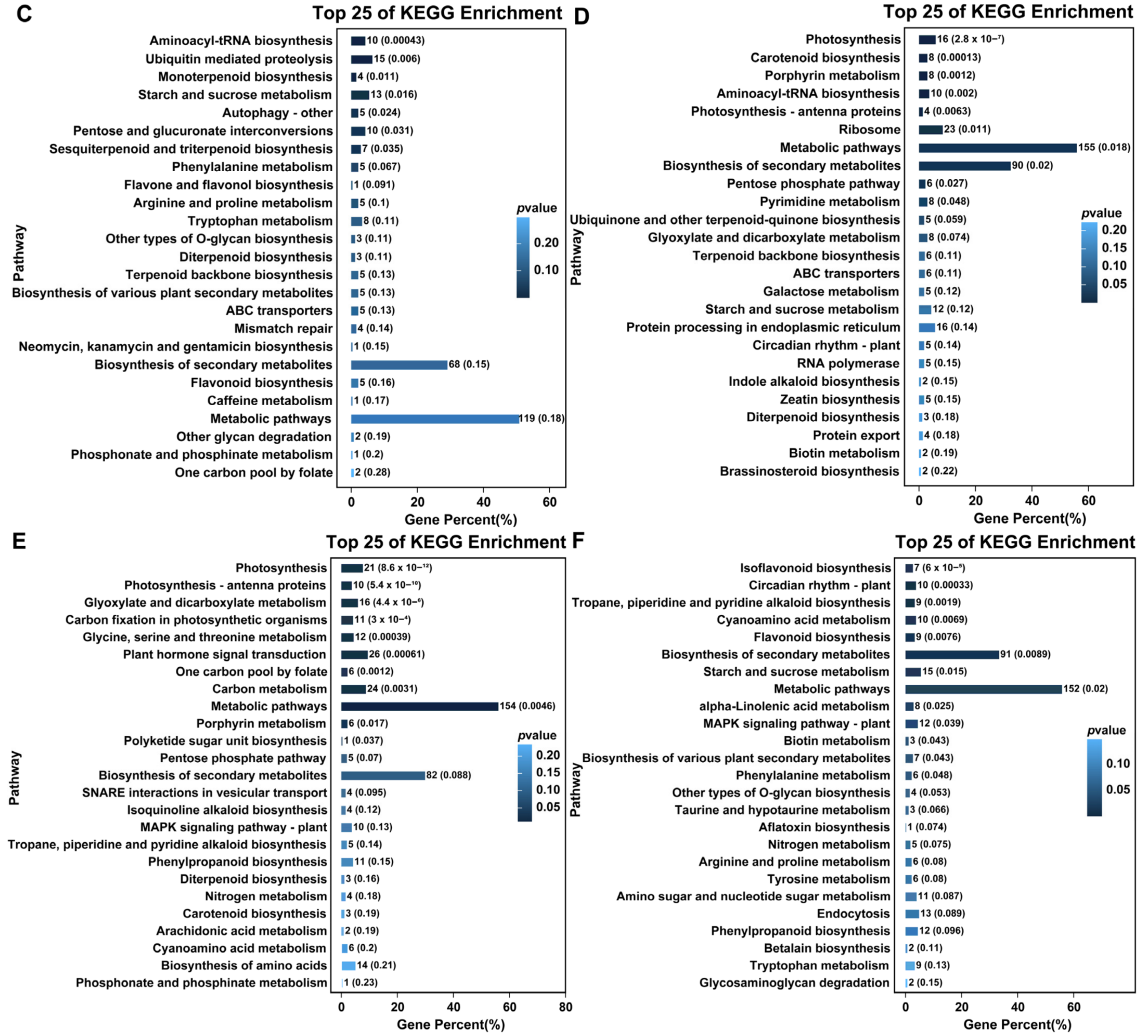
The KEGG enrichment pathways in profile 0, profile 1, profile 2, profile 3, profile 4, and profile 5 in ‘SO4’ and ‘Beida’ grapevine rootstocks (**A**–**F**).

Further trend analysis categorized the DEGs into distinct expression profiles (Profiles 0 to 19, Supplementary Figure S5) based on their response trajectories over the 12-day stress period. The DEGs in Profile 2, Profile 3, Profile 4, and Profile 5 were found to align with the trends of alterations under tolerance conditions. Profiles 0 and 1 indicated a negative trend regarding susceptibility conditions in grape rootstocks subjected to salt stress (Figure 9A–C). Among these, Profile 0 specifically captured genes associated with active tolerance mechanisms. KEGG analysis of Profile 0 revealed an abundance of genes regulating defense pathways, including sesquiterpenoid and triterpenoid biosynthesis, fatty acid metabolism, and ER protein processing. Meanwhile, Profiles 1 through 5, which captured broader stress-responsive gene expression, were heavily enriched in pathways driving physiological adaptation, including phenylpropanoid biosynthesis, starch and sucrose metabolism, and ubiquitin-mediated proteolysis (Figure 9D–F). These profiles underscore the heavy metabolic restructuring required to generate the osmotic adjustments (like soluble sugar accumulation) observed physiologically.

### 2.6. Activation of Specific TF Families

**Figure 10:**
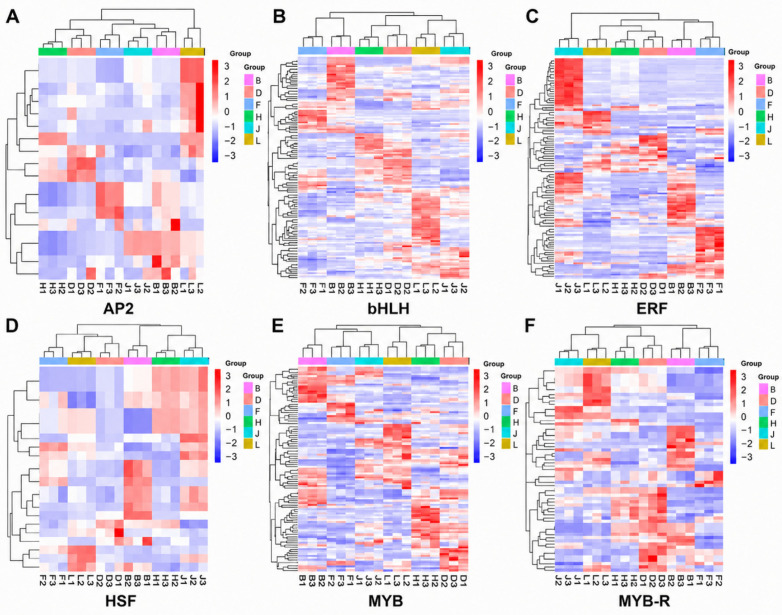
Heatmap of differentially expressed transcription factors in ‘SO4’, and ‘Beida’ grapevine rootstock under salt stress (NaCl; 100 mmol L^−1^). (**A**) Activating protein-2 (AP2), (**B**) basic helix loop helix (bHLH), (**C**) ethylene response factor (ERF), (**D**) heat shock factor (HSF), (**E**) myeloblastosis viral oncogene homolog (MYB), and (**F**) myeloblastosis viral oncogene homolog response (MYB-R). From (**A**–**F**) represents the enriched TFs between 0, 6, and 12 d of stress duration adjacent time (B1, B2, B3: 0d), (D1, D2, D3: 6d), and (F1, F2, F3: 12 d) in ‘SO4’; similarly, there are (H1, H2, H3: 0d), (J1, J2, J3: 6d), and (L1, L2, L3: 12 d) in ‘Beida’ grapevine rootstocks.

TFs are the main controllers of how plants respond to stress. Our transcriptome data revealed 676 differentially expressed TFs spanning seven principal gene families: basic helix loop helix (bHLH), activating protein-2 (AP2), ethylene response factor (ERF), heat shock factor (HSF), WRKY, myeloblastosis viral oncogene homolog (MYB), and MYB-related (Figure 10A–F and Supplementary Figure S6). The expression patterns of these TFs suggested distinct regulatory roles. During salt stress, the AP2 and HSF family TFs were mostly down-regulated. The MYB, WRKY, and ERF families, which are usually linked to signaling for ROS scavenging and the biosynthesis of secondary metabolites, were highly up-regulated in both rootstocks. The robust stimulation of these particular transcription factor families likely facilitates the subsequent activation of antioxidant enzymes (SOD, POD, CAT, APX) and the extensive transcriptome reprogramming necessary for grapevine salt tolerance.

## 3. Discussion

Integrating our physiological and transcriptomic data, this study demonstrates that ‘SO4’ exhibits comparatively greater resilience than ‘Beida’ through a highly coordinated defense strategy. The stronger retention of photosynthetic pigments in ‘SO4’ (Figure 1) suggests that this genotype effectively mitigates chlorophyll degradation, a common consequence of salt-induced oxidative damage [28,36,37,38].

At the biochemical level, the contrasting responses of the two rootstocks illuminate distinct coping mechanisms. Plants experiencing salt stress undergo coordinated physiological and biochemical adjustments to counter osmotic imbalance and cellular damage [39,40]. A central consequence is oxidative injury, reflected by elevated levels of ROS, including H_2_O_2_, and increased lipid peroxidation, commonly quantified as MDA [40]. To alleviate these effects, plants accumulate compatible solutes such as proline, soluble carbohydrates, and soluble proteins, which function as osmolytes to stabilize cellular structures, preserve enzyme activity, and sustain water uptake [40,41,42]. However, some species exhibit reduced sugar and protein contents at high salinity. Yağcı [43] reported that NaCl at 150 mM markedly suppressed sugar metabolism (glucose and fructose) in *Vitis vinifera* cv. ‘Öküzgözü’. In cucumber genotypes tested in vitro and in vivo, soluble sugars and protein both decreased with increasing salinity across tolerant and sensitive lines [44], indicating species- and genotype-dependent responses [45], and salt-tolerant cultivars often display lower MDA and H_2_O_2_ levels together with enhanced accumulation of osmolytes compared with their sensitive counterparts [46]. These contrasting patterns across genotypes are informative indicators of salt tolerance capacity and are consistent with the differential responses observed between ‘SO4’ and ‘Beida’ [39].

In our study, increasing salt levels led to higher soluble sugar, soluble protein, proline, MDA, and H_2_O_2_ contents, as well as enhanced antioxidant activities in both ‘SO4’ and ‘Beida’ rootstocks, in agreement with previous observations that salt-stressed plants activate similar metabolic responses [47,48]. The accumulation of compatible solutes is a key adaptive strategy under high salinity, contributing to osmotic adjustment and protection of cellular components [48]. Notably, ‘SO4’ accumulated more free proline than ‘Beida’ under salt stress (Figure 2C), which is consistent with the high osmotic adjustment capacity previously reported in ‘SO4’ [49], which is consistent with greater osmotic adjustment in the tolerant genotype. Concurrent increases in MDA and H_2_O_2_ indicate salt-induced oxidative stress and excessive ROS production, a typical feature of salinity damage in grapevine and other crops [50]. Interestingly, ‘SO4’ exhibited higher absolute levels of H_2_O_2_ and MDA than ‘Beida’. While traditionally viewed as direct biomarkers of oxidative injury, this paradoxical accumulation in the tolerant genotype suggests that these molecules may function as regulated signaling intermediates involved in coordinating the active stress acclimation response. The rapid, sustained generation of H_2_O_2_ in ‘SO4’ likely serves as an amplified early-warning signal that initiates systemic acquired acclimation. As a relatively stable secondary messenger, H_2_O_2_ acts upstream to activate redox-sensitive transcription factors (e.g., WRKY, MYB, and ERF families) and provides the necessary steady-state substrate to continuously prime and upregulate its enzymatic antioxidant machinery (SOD, CAT, and APX). Similarly, the elevated MDA observed in ‘SO4’ likely represents a critical membrane signaling event. Recent advances in plant lipidomics suggest that transient lipid peroxidation physically perturbs membrane microdomains, triggering the release of lipid precursors necessary for the biosynthesis of defense hormones (like jasmonic acid) and driving the massive metabolic shift toward secondary metabolite synthesis (e.g., flavonoids and phenylpropanoids) observed in our transcriptomic data. Thus, the high H_2_O_2_ and MDA in ‘SO4’ act as catalysts for a highly active signal transduction network that ultimately yields superior osmotic adjustment and ionic homeostasis [51,52,53,54,55]. Similar coordinated activation of osmolyte accumulation and antioxidant defenses has been reported as a hallmark of plant salt tolerance [48,56]. The stronger magnitude of these biochemical responses in ‘SO4’ compared with ‘Beida’ is therefore consistent with its higher salt tolerance and supports the notion that tolerant cultivars deploy more robust defense mechanisms [29,57].

At the transcriptomic level, pairwise DEG analyses showed that the salt-tolerant grapevine genotype possessed more DEGs than the salt-susceptible genotype, similar to patterns described for salt- and drought-stressed crops where tolerant genotypes exhibit broader transcriptional reprogramming [23,58,59]. These differences may be partly related to leaf morphological traits, as tolerant genotypes often develop broader leaves compared with the thinner leaves of susceptible genotypes, which can influence both ion distribution and stress perception. Muthusamy et al. [60] noted that leaves are primary organs involved in salt stress responses, and our observation that salt-tolerant leaves had more DEGs than susceptible ones supports this view. Among the significantly enriched pathways, 72 DEGs in salt-stressed leaves were associated with key processes such as carbon fixation, circadian rhythm, protein processing in the endoplasmic reticulum, and hormone-mediated signaling, in line with previous reports of abiotic stress-responsive pathways [61,62,63]. GO and KEGG analyses further revealed that alkaloid biosynthesis, unsaturated fatty acid biosynthesis, glycerophospholipid biosynthesis, linoleic acid metabolism, porphyrin metabolism, carbon fixation, and plant hormone signal transduction were differentially modulated between ‘SO4’ and ‘Beida’. Rather than implying complete conservation of salt tolerance mechanisms, these results suggest that both shared and genotype-specific signaling networks are engaged, with tolerant ‘SO4’ exhibiting more extensive remodeling of metabolic and signaling pathways [59]. While several of these DEGs (e.g., general ROS scavengers) have been previously implicated in salinity responses, their specific coordinated expression patterns within the MEblack module and their comparative dynamics between ‘SO4’ and ‘Beida’ represent novel findings in grapevine rootstock biology.

WGCNA identified five modules (MEblack, MEblue, MEyellow, MEgreen, and MEgrey) that were strongly associated with salt tolerance differences between the rootstocks. Among these, MEblack was positively correlated (upregulated) in ‘SO4’ but negatively correlated in ‘Beida’ under salt stress, and it encompassed the largest number of DEGs (274), indicating that genes within this module may be central to the enhanced tolerance of ‘SO4’. Furthermore, intramodular analysis of the MEblack module was conducted to identify the key regulatory nodes driving the ‘SO4’ salt tolerance phenotype. By calculating intramodular connectivity (Degree) and module membership (kME), we identified specific hub genes that exhibited both high network centrality and high gene significance (GS) for salt tolerance. The gene with the highest overall connectivity (Degree = 848.4) and strongest trait association (GS = 0.895) was *Vitvi01g00735* (kME = 0.952). The MEblack module is significantly enriched in plant hormone signal transduction and secondary metabolite biosynthesis. The high module membership, gene significance, and intramodular connectivity of *Vitvi01g00735/VvBCA2* identify it as a strong candidate hub associated with the coordinated salinity response of the MEblack module. Additionally, *Vitvi07g02043* (kME = 0.964, GS = 0.851) and *Vitvi14g00619* (kME = 0.962, GS = 0.861) were identified as secondary hub nodes. Although the functions of several neighbouring and unresolved gene models require experimental validation, the strong module membership and trait associations of *VvBCA2* and *VvLCB1* support their prioritization as candidate salinity-associated hubs, and their strong topological positions and high trait significance indicate they are intimately involved in the salt stress response network. The coordinated high connectivity of these specific hub genes within the hormone and secondary metabolism clusters provides a concrete, network-based explanation for the ‘SO4’ tolerance phenotype, shifting the focus from broad family-level classifications to specific, highly interconnected regulatory candidates. This pattern is consistent with previous reports of cultivar-dependent coexpression modules underlying differential stress responses [63,64]. In *Astragalus cicer* L., for example, the MEturquoise, MEgreen, MEblue, and MEpink modules were closely linked to salinity stress tolerance [65], and similar genotype-specific modules have been noted in diverse crops because of genetic diversity and evolutionary history [66]. Our observation that module eigengenes summarize DEGs and correlate with expression profiles within each module agrees with previous findings on the utility of eigengene-based network summaries to capture coordinated stress-responsive transcriptional patterns [67]. Collectively, these results connect the more favorable physiological performance of ‘SO4’ with the activation of distinct salt-responsive coexpression networks.

The *VvBCA2*-centred network suggests that carbon metabolism may be coordinated with redox regulation, protein homeostasis, defence signalling, and osmotic adjustment. Carbonic anhydrases catalyse the reversible conversion of CO_2_ and bicarbonate and contribute to inorganic-carbon availability and cellular pH regulation [68]. The connections of *VvBCA2* with *NADK1-like*, *TRX-X*, *LOX1*, *CRT3*, *PDIL1-1*, *CML23-like*, and *OSCA1-like* indicate potential coordination between carbon metabolism, NADP(H)-dependent redox balance, protein quality control, oxylipin metabolism, and calcium-mediated osmotic signalling. This coordinated neighbourhood may help sustain metabolic activity and stress-responsive signalling in ‘SO4’ under salinity. Nevertheless, these relationships are based on co-expression and do not establish direct regulatory or biochemical interactions.

The *VvLCB1*-centred network was more strongly associated with membrane-lipid metabolism, cell-wall modification, one-carbon metabolism, and stress signalling. LCB1 is an essential component of serine palmitoyltransferase, which catalyses the first committed step of sphingolipid biosynthesis [69,70]. The association of *VvLCB1* with *HPL1*, *ACX1*, *KCS9-like*, and *GDSL esterase 1* supports coordinated regulation of sphingolipid, oxylipin, fatty-acid, and membrane-remodelling processes. Its strong connections with *PP2C76*, *SAM synthase 2*, and *OSCA1-like* further connect this neighbourhood with ABA-related protein phosphorylation, S-adenosylmethionine-dependent metabolic regulation, and hyperosmotic calcium signalling. These processes may contribute to membrane stability, cell-wall adjustment, and signal transduction in the salt-tolerant rootstock.

The presence of *PP2C76*, *SAM synthase 2*, and *OSCA1-like* connects the *VvLCB1* neighbourhood with established components of plant salinity signalling. Specific plant PP2Cs regulate ABA-dependent salt responses, and overexpression of *AtPP2CG1* enhances salt tolerance in *Arabidopsis* [71]. Similarly, overexpression of tomato *SlSAMS1* improves salt tolerance and influences DNA methylation of *SlGI*, supporting a relationship between S-adenosylmethionine metabolism, epigenetic regulation, and salinity responses [72]. *OSCA1* is a hyperosmolality-responsive calcium-permeable channel required for osmotic-stress-induced calcium signalling in *Arabidopsis* [73]. The grape homologues identified here may perform related functions, although this requires experimental confirmation.

The limited overlap between the two networks indicates that *VvBCA2* and *VvLCB1* represent complementary rather than redundant co-expression neighbourhoods. The *VvBCA2* network was characterized mainly by genes associated with redox regulation, protein folding, defence, and hormone-related signalling, whereas the *VvLCB1* network contained more genes related to cell-wall remodelling, methyl metabolism, membrane signalling, and lipid turnover. Their shared association with *OSCA1-like*, *AER-like*, and *KCS9-like* suggests convergence through osmotic calcium signalling, reactive-carbonyl detoxification, and membrane-lipid adaptation [73]. The shared *DUF1666* gene and the strongly connected unresolved genes, including *Vitvi07g04117*, *Vitvi08g04383*, and *Vitvi13g04170*, represent particularly important candidates for expression validation and functional characterization.

A time-course analysis of DEG expression patterns identified six distinct profiles (Profiles 0–5), reflecting dynamic transcriptional trajectories linked to tolerance and susceptibility. Profiles 2, 3, 4, and 5 were positively associated with salinity tolerance, whereas Profiles 0 and 1 showed negative associations and were linked to susceptible responses. KEGG annotation indicated that genes in these profiles participate in diverse pathways, including metabolic processes, plant–pathogen interactions, biosynthesis of secondary metabolites, and sesquiterpenoid and triterpenoid biosynthesis [74]. These dynamic expression clusters suggest that salt tolerance in ‘SO4’ involves both early and sustained transcriptional adjustments in metabolism and defense-related pathways, which are less pronounced or differently regulated in ‘Beida’. When considered alongside the biochemical data, the profiles associated with tolerance coincide with enhanced osmolyte accumulation and antioxidant enzyme activity, reinforcing the tight coordination between physiological and transcriptomic responses in the tolerant rootstock.

**Figure 11:**
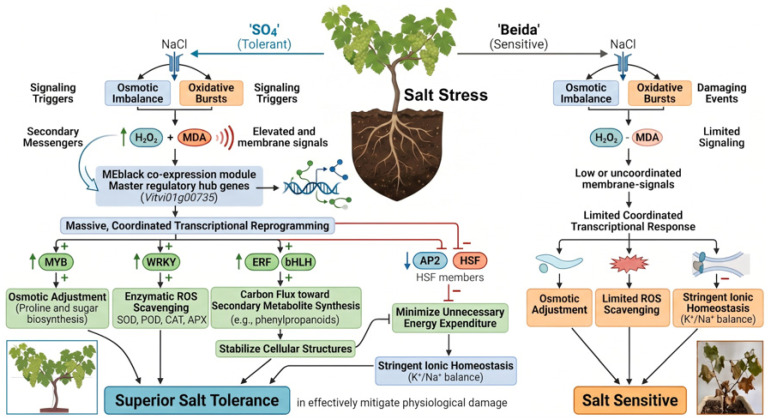
Proposed regulatory model illustrating the integrated morphophysiological and transcriptomic mechanisms driving salt tolerance in the ‘SO4’ grapevine rootstock. Upon sodium chloride (NaCl) exposure, osmotic imbalance and oxidative bursts are perceived not merely as damaging events, but as critical signaling triggers. In the tolerant ‘SO4’ genotype, elevated levels of hydrogen peroxide (H_2_O_2_) and malondialdehyde (MDA) act as indispensable early-warning secondary messengers and membrane signals. This amplified signaling cascade activates the highly connected ‘MEblack’ co-expression module, orchestrated by master regulatory hub genes (primarily *Vitvi01g00735*). These hub genes drive a massive, coordinated transcriptional reprogramming event. Upregulated myeloblastosis viral oncogene homolog (MYB) and WRKY transcription factors (TFs) stimulate robust osmotic adjustment (proline and sugar biosynthesis) and continuous enzymatic reactive oxygen species (ROS) scavenging (SOD: superoxide dismutase, POD: peroxidase, CAT: catalase, and APX: ascorbate peroxidase). Concurrently, ethylene response factor (ERF) and basic helix loop helix (bHLH) TFs direct carbon flux toward secondary metabolite synthesis (e.g., phenylpropanoids) to stabilize cellular structures, while specific activating protein-2 (AP2) and heat shock factor (HSF) members are strategically downregulated to minimize unnecessary energy expenditure. Together with stringent ionic homeostasis (potassium/sodium: K^+^/Na^+^ balance), this highly coordinated molecular and biochemical network effectively mitigates physiological damage, culminating in superior salt tolerance in the ‘SO4’ rootstock compared to the sensitive ‘Beida’. Green upward arrows indicate increased levels, enhanced expression, or positive regulation, whereas downward arrows indicate decreased expression or suppression. (+) symbols denote positive regulatory effects. (−) symbols denote negative regulatory effects. Different colors are used to distinguish signaling molecules, regulatory modules, and transcription factors.

We propose a regulatory framework describing how the identified TFs coordinate these defenses in ‘SO4’ (Figure 11). Under salt stress, early ROS and osmotic signals likely trigger the upregulation of MYB and WRKY TFs, which subsequently activate downstream genes responsible for osmolyte biosynthesis (proline and soluble sugars) and antioxidant enzyme activity (SOD, POD, CAT, and APX). Concurrently, ERF and bHLH TFs modulate secondary metabolic pathways (e.g., phenylpropanoids) to stabilize cellular membranes, while AP2/ERF and HSF families fine-tune the overall stress response, with certain members being down-regulated to prevent unnecessary energy expenditure [29,34,59,75].

Consistent with these findings, our study revealed that most differentially represented TFs were enriched in ‘SO4’ relative to ‘Beida’ under salt stress, indicating a more active transcriptional regulatory network in the tolerant rootstock. Joshi et al. [76] also emphasized that TF families are critical determinants of abiotic stress adaptation in crops. Taken together, our morphophysiological and transcriptomic analyses support a model in which ‘SO4’ tolerance is associated with enhanced osmotic adjustment, reduced oxidative damage, and the activation of specific stress-responsive modules and TF networks, whereas ‘Beida’ exhibits a more limited and less coordinated response. As rootstocks primarily influence scion performance, the robust molecular defenses identified in ‘SO4’ leaves likely translate to improved scion water and nutrient status, ultimately sustaining growth and berry yield under saline field conditions [77,78,79]. Our research therefore contributes to a more integrated understanding of how morphophysiological adaptations are linked with genomic responses in grapevine cuttings exposed to salt stress. Future studies employing transgenic or gene-editing approaches (e.g., CRISPR/Cas9) will be necessary to functionally validate the causal roles of the identified hub genes and TFs.

## 4. Materials and Methods

### 4.1. Experimental Conditions, Salt Treatments, and Sampling Layout

The experiment conducted in 2023 used one-year-old, non-grafting rootstocks of two grapevine rootstocks, ‘SO4’ (*Vitis riparia × Vitis berlandieri*) and ‘Beida’ (*Vitis riparia × Vitis labrusca*), as the plant material (Minkang Nursery Stock, Co., Ltd., Qinhuangdao, China). The cuttings of two grapevine rootstocks were grown in pots (50 cm length × 25 cm width × 30 cm deep) in a greenhouse at Bai Ma Base Research Field, Nanjing Agricultural University, Nanjing, Jiangsu, China, at temperature 20–25, 65% humidity vs. a 16 h light and 8 h dark period. We used a mixture of peat, vermiculite, and perlite (3:1:1, *v*/*v*/*v*) as the cultivation soil. The total number of pots was 16 per rootstock, with 10 cuttings in each pot. The cuttings were fertilized every 6 days [80], with the following contents: 85.31 mg of N, 7.52 mg of P, 104.75 mg of K, 97.64 mg of Ca, 23.68 mg of Mg, 11.54 mg of S, 2.68 mg of Fe, 0.03 mg of Cu, 0.13 mg of Zn, 0.11 mg of Mn, 0.27 mg of B, 0.05 mg of Mo, and 0.01 mg of Ni per L. Irrigation was done every three days for a month until they developed healthy roots and leaves.

One month after transplantation, uniformly growing cuttings with comparable shoot length and vigor were randomly selected and subjected to salt stress treatments under the controlled conditions, as mentioned previously. There were two types of irrigation: a control group (SO4-CK and Beida-CK) that got distilled water and a salt-stress group (SO4-NaCL and Beida-NaCl) that got a 100 mmol L^−1^ NaCl solution [29]. A concentration of 100 mmol L^−1^ NaCl was selected because it represents a moderate-to-severe salinity level commonly observed in affected viticultural soils, effectively inducing physiological stress without causing immediate lethality, thereby allowing the observation of adaptive molecular mechanisms. Each treatment consisted of three independent replicates. To ensure uniform stress, a predetermined, constant volume of the 100 mmol L^−1^ NaCl solution was applied to each pot every three days, maintaining consistent soil moisture and salinity levels across all replicates without causing waterlogging or leaching. In total, 240 cuttings were used for the experiment, comprising 120 cuttings for each of the two rootstocks (SO4 and Beida). The cuttings were planted into pots, with five cuttings planted per pot, resulting in 24 pots per rootstock. The plants were irrigated every three days throughout a 12-day salt stress period. Leaf samples were collected at 0, 6, and 12 days after treatment initiation. Specifically, the fourth fully expanded leaf from the apex was harvested, immediately frozen in liquid nitrogen, and stored at −80 °C for subsequent physiological, biochemical, and RNA-seq analyses. Three biological replicates were included for each sampling time point.

### 4.2. Assessment of Leaf Chlorophyll Pigment Content

The concentrations of chlorophyll *a*, chlorophyll *b*, total chlorophyll, and carotenoids were measured using 0.25 g of fresh leaf tissue. After homogenizing the tissue in 10 mL of 80% acetone, it was centrifuged for 10 min at 4 °C at 6000 rpm. The supernatant obtained was then subjected to a spectrophotometer (Analytik Jena Spekol 2000, Jena, Germany). Absorbance readings were recorded at 663 nm for chlorophyll *a*, 645 nm for chlorophyll *b*, and 470 nm for carotenoids using a spectrophotometer, and the results were calculated according to Shafqat et al. [81]. The readings were expressed as mg g^−1^ FW (Fresh Weight).

### 4.3. Determination of Mineral Ions

The mineral element analysis of leaves from treated and control plants for ‘SO4’ and ‘Beida’ rootstocks involved drying the samples in a thermostatic oven at 80 °C. Subsequently, the desiccated substance is meticulously pulverized utilizing a mortar and pestle. Subsequently, 0.2 g of the resultant powder was digested with 5 mL of sulfuric acid (H_2_SO_4_). Subsequently, the amounts of nutritional elements, specifically Na^+^ and potassium (K^+^), were determined using an inductively coupled plasma atomic emission spectrometer, following the approach outlined by Peng et al. [82]. Finally, the K^+^/Na^+^ ratio for each sample was calculated by dividing the K^+^ content by the Na^+^ content.

### 4.4. Determination of Antioxidant Enzyme Activities

To extract enzymes, 0.5 g of leaf powder was homogenized in 4 mL of extraction buffer consisting of 50 mM potassium phosphate, 1 mM ethylenediaminetetraacetic acid, 1% polyvinylpyrrolidone, 1 mM dithiothreitol, and 1 mM phenylmethylsulfonyl fluoride at pH 7.8. The extractions underwent centrifugation at 15,000× *g* for 30 min at 4 °C [83]. The supernatant was collected and stored at −80 °C for the analysis of SOD, CAT, APX, and POD. The activity of SOD was assessed by observing the inhibition of the photochemical reduction of nitro blue tetrazolium (NBT), following the methodology established by Giannopolitis and Ries [84]. A single unit of SOD activity is defined as the quantity of enzyme necessary to achieve 50% inhibition of NBT reduction, as measured at 560 nm using a UV spectrophotometer. SOD activity was quantified in units mg^−1^ protein. The POD activity was assessed using guaiacol as the substrate in a total volume of 3 mL. The reaction mixture comprised 50 mM potassium phosphate buffer (pH 6.1), 1% (*w*/*v*) guaiacol, 0.4% (*v*/*v*) H_2_O_2_, and enzyme extract. An elevation in absorbance resulting from the oxidation of guaiacol (E = 25.5 mM^−1^ cm^−1^) was recorded at 470 nm using a UV spectrophotometer, and the findings were represented in units mg^−1^ protein [85]. CAT activity was assessed in 50 mM potassium phosphate at pH 6.6 and 24 °C. The reaction commenced with the addition of H_2_O_2_ to a concentration of 10 mM. Enzyme activity, namely oxygen generation, was monitored for 2 to 4 min, and the readings were recorded using a UV spectrophotometer at 240 nm. The results were quantified as units mg^−1^ H_2_O_2_ [86]. APX activity was induced in a mixture of phosphate buffer (50 mM, pH 7.0), 0.1 mM EDTA, 0.1 mM H_2_O_2_, and 0.5 mM ascorbate. APX was measured by tracking the H_2_O_2_-dependent oxidation of ascorbate, which was detected as a drop in absorbance at 290 nm [87].

### 4.5. Determination of Soluble Sugar Content, Proline, and Protein

The method outlined by Iqbal et al. [20] was used to determine soluble sugar and proline contents. To measured soluble sugar content, 0.10 mL of the alcoholic extract was mixed with 3 mL of freshly prepared anthrone reagent, containing 150 mg of anthrone dissolved in 100 mL of 72% sulfuric acid. The final mixture was incubated for 10 min at 90 °C in a steam bath and cooled to room temperature. Absorbance was determined at 625 nm by a spectrophotometer. Proline content was determined using the acid-ninhydrin method. Fresh leaf tissue (0.5 g) was homogenized in 5 mL of 95% ethanol, and the residue was re-extracted with 5 mL of 70% ethanol. The combined supernatants were clarified by centrifugation at 15,000 rpm for 15 min. A 1 mL aliquot of the resulting extract was diluted with 9 mL of distilled water and reacted with 5 mL of glacial acetic acid and 5 mL of acid-ninhydrin reagent. The mixture was incubated at 90 °C for 45 min. After cooling, the proline-ninhydrin chromophore was partitioned into 4 mL of toluene. The absorbance of the toluene layer was measured spectrophotometrically at 515 nm. To determine protein, 0.5 g sample of fresh leaf tissue was pulverized in a mortar with 10 mL of filtered water and then quantitatively transferred to test tubes. In total, 1 mL of 10% trichloroacetic acid was introduced, followed by the placement of the tubes in an ice bath for 10 min. The supernatant was isolated from the precipitation and subjected to centrifugation at 5000 rpm for 10 min at 4 °C. The precipitate was cleared in 20 mL of 0.1 N sodium hydroxide to solubilize the protein, and the volume was rounded to the nearest whole number, according to Lowry et al. [88].

### 4.6. Determination of MDA and H_2_O_2_

MDA was quantified by combining 0.2 g of fresh plant leaf extract with 2 mL of 0.5% trichloroacetic acid and centrifuging the mixture for 15 min at 12,000 rpm. Subsequently, 1 mL of the supernatant was combined with 4 mL of 0.5% thiobarbituric acid in a centrifuge tube, which was then subjected to heating in a water bath at 95 °C for 30 min. The test tubes were subsequently chilled on ice, and the optical density of the reaction mixture was assessed at absorbance wavelengths of 532 nm and 600 nm using a spectrophotometer, according to the methodology outlined by Heath and Packer [89]. The quantification of H_2_O_2_ was conducted via ferrous oxidation with Xylenol Orange (FOX) reagent, according to the methodology outlined by Rhee et al. [90]. A fresh leaf sample (0.5 g) was pulverized in liquid nitrogen with a mortar and pestle, incorporating 2 mL of cold acetone. The aggregated mixture was subjected to centrifugation for 20 min at 10,000 rpm. Subsequently, 100 μL of the resultant supernatant was homogenized for 1 h in the presence of FOX reagent, while absorbance readings at 560 nm were obtained using a spectrophotometer to quantify the relative concentration, according to Abdelrady et al. [91].

### 4.7. RNA Isolation, cDNA Synthesis, and Transcriptome Sequencing Analysis

RNA was extracted from the leaves of the ‘SO4’ and ‘Beida’ grapevines subjected to salt stress for 0, 6, and 12 days, using three distinct samples for each time interval. RNA was extracted using the cetyltrimethylammonium bromide (CTAB) method [92]. The RNA extracted from both grapevine rootstocks (leaves) was treated with RNase-free DNase I at 25 °C for 15 min to eliminate residual DNA. The concentration and purity of the extracted RNA were subsequently measured using a NanoDrop™ 2000 spectrophotometer (Thermo Fisher Scientific, Wilmington, DE, USA). The integrity of total RNA was assessed using a Bioanalyzer 2100 system (Agilent Technologies, Waldbronn, Germany). The RNA library for each sample was labelled and constructed using a high-throughput Illumina NovaSeq 6000 (Gene Denovo Biotechnology Company Ltd., Guangzhou, China).

For RNA sequencing, 18 samples from the control group and salt stress treatments at 0, 6, and 12 days, comprising three independent biological replicates and nine comparison groups, were analyzed to evaluate tolerance levels and gene expression values. There were 6 experimental sample groups (B, D, F, H, J, and L). We conducted 9 specific pairwise comparisons between these 6 groups to evaluate the treatment effects. These 9 comparisons are B vs. D (0d_6d of NaCl), B vs. F (0d_12d of NaCl), B vs. H (0d_0d of NaCl), D vs. F (6d_12d of NaCl), D vs. J (6d_6d of NaCl), J vs. L (6d_12d of NaCl), F vs. L (12d_12d of NaCl), H vs. J (0d_6d of NaCl), and H vs. L (0d_12d of NaCl).

The library sequencing was completed using an Illumina HiSeq X platform, and raw data were considered as raw reads. Additionally, the clean reads were obtained by removing adapter sequences and low-quality reads, and then the biological sequencing data was extracted using a custom Perl script that required a base pair quality of Q ≥ 20. The filtered reads were aligned to the reference genome (https://plants.ensembl.org/Vitis_vinifera/Info/Index?db=core; accessed on 10 March 2024) using HISAT2 software (version: 2.2.1), which was used to calculate the number of fragments per kilobase per exon (FPKM) per million tagged reads to estimate how much of each transcript was present with a 95% confidence level. DESeq2 was used to determine DEGs with the screening criteria | log_2_FC (fold change) | ≥ 1 and false discovery rate (FDR) < 0.05.

To obtain more detailed information on the DEGs, we performed an enrichment analysis using the R program (version: 3.6.3) [93]. The OmicShare web tool (https://www.omicshare.com/tools/Home/Soft/getsoft; accessed on 20 April 2024) was used to construct Gene Ontology (GO; http://geneontology.org/) and Kyoto Encyclopedia of Genes and Genomes (KEGG; https://www.genome.jp/kegg/) database annotation pathways of all the DEGs [94]. To determine the significant enrichment analysis of KEGG-related terms, we use the threshold of *padj* < 0.05. Each sample contained three independent biological replications.

### 4.8. Construction of WGCNA

To find gene expression networks, we used WGCNA (version: 1.74) with FPKM values from both plant types to identify groups of genes that work together under salinity stress [63,95]. Prior to network construction, genes and samples with excessive missing values were identified using the goodSamplesGenes function, and genes or samples flagged as unsuitable were removed from the expression matrix. Because none of the tested powers reached the predefined scale-free topology fit criterion of approximately (R^2^ ≥ 0.85), an empirical soft-thresholding power of (β = 18) was selected based on the scale-free topology and mean-connectivity analyses.

Using this power, a signed weighted adjacency matrix was constructed by transforming pairwise Pearson correlations between gene-expression profiles into continuous co-expression connection strengths. The adjacency matrix was subsequently transformed into a topological overlap matrix (TOM), which was used as the input for average-linkage hierarchical clustering. Gene modules were identified from the resulting dendrogram using the dynamic tree-cut algorithm implemented in blockwiseModules with a signed network type. The overall expression pattern of each module was summarized by its module eigengene, defined as the first principal component of the module’s expression matrix. Module eigengenes were then correlated with salinity-related physiological traits to identify modules significantly associated with salt stress.

Genes within the salinity-associated modules were evaluated using module membership (kME), gene significance for salinity-related traits, and weighted intramodular connectivity. Within the MEblack module, *Vitvi01g00735/VvBCA2* and *Vitvi07g02043/VvLCB1* were selected as biologically relevant central genes based on their high kME values, strong gene significance, intramodular connectivity, and functional annotations. For each central gene, all direct within-module connections were extracted from the signed adjacency matrix and ranked in descending order according to their soft-thresholded adjacency weights. The 30 genes showing the highest adjacency weights with each central gene were retained to construct the hub-centred co-expression networks, following the network-selection approach described by Chen et al. [64].

In the resulting networks, edge width and neighbouring-node size were scaled according to the adjacency weight connecting each gene to the central gene, whereas the central gene was displayed at a fixed larger size. Node colors represented broad functional categories, while node shapes and border widths indicated annotation confidence and unresolved or weakly mapped gene models. kME was used to support central-gene selection. Heatmaps of module–trait relationships and gene expression patterns were generated using TBtools (v 2.450).

### 4.9. Validation of RNA-Seq by qPCR Analysis

This study includes Supplementary Table S1, which presents the specific primer list developed using the Primer 3 Plus web tool and the National Center for Biotechnology Information (NCBI) resources (https://primer3.org/ accessed on 20 April 2024 vs. https://www.ncbi.nlm.nih.gov/). Quantitative real-time polymerase chain reaction (RT-qPCR) was conducted to confirm the relative expression of selected genes from ‘SO4’ and ‘Beida’ grapevine rootstocks subjected to salt stress. RNA extraction from the leaves of both rootstocks was conducted using the CTAB method, as described by Dong et al. [96]. The cDNA was synthesized with HifairII First Strand cDNA Synthesis SuperMix for RT-qPCR (Yaseen, Shanghai, China), and RT-qPCR was performed using a 2 × TSINGKE^®^ Master RT-qPCR Mix (SYBR Green I) (Beijing Tsingke Biotechnology Co., Ltd., Beijing, China). The total volume of the reaction mixtures was 10 μL, comprising 5 μL of SYBR Green Supermix, 0.3 μL of primer (10 μM), 2 μL of cDNA, and 2.4 μL of RNase-free water. The RT-qPCR cycle parameters included a predenaturation step at 95 °C for 2 min, followed by denaturation at 95 °C for 10 s, primer annealing at 60 °C for 40 s, and extension for 40 cycles, concluding with denaturation. The grapevine *VvActin* gene was used as the internal reference control to normalize the expression levels of the target genes. The relative expression of treated samples compared to the control group was determined using the 2^−∆∆CT^ method [97].

### 4.10. Statistical Analysis

The experimental data underwent a two-way analysis of variance (ANOVA). Each treatment had three technical replications and was presented as mean ± standard deviation (SD). Tukey’s test assessment was applied to determine whether treatment means from repeated trials differed significantly at *p* < 0.05 value. The graphs and heatmaps have been created using OriginPro 2021, OmicShare website (https://www.omicshare.com/tools/, accessed on 10 May 2024), and TBtools software (version: 2.475).

## 5. Conclusions

This study provides valuable insights into the complex physiological and molecular mechanisms that regulate salt stress tolerance in grapevine rootstocks. The combined biochemical and transcriptomic analyses indicate that the greater resilience of ‘SO4’ is associated with a highly coordinated defense response involving osmotic adjustment, reflected by the accumulation of soluble carbohydrates and proline, and rapid activation of antioxidant enzymes, including SOD, POD, CAT, and APX, to mitigate salinity-induced oxidative stress. WGCNA further identified the MEblack module as a major salinity-associated network and prioritized *Vitvi01g00735/VvBCA2* and *Vitvi07g02043/VvLCB1* as candidate hub genes linked to complementary processes involving redox homeostasis, protein quality control, cell-wall remodelling, osmotic signalling, and membrane-lipid metabolism. Their shared connections with *OSCA1-like*, *AER-like*, *KCS9-like*, and *DUF1666* also highlight potential points of convergence and provide additional targets for functional investigation. In conclusion, these findings support our initial hypothesis that ‘SO4’ exhibits a more robust and coordinated molecular response compared to ‘Beida’. Rather than claiming a complete mechanism, this study provides a comprehensive transcriptomic landscape and proposes a regulatory framework wherein specific TF families (MYB, WRKY, ERF) coordinate osmotic adjustment and antioxidant defenses. Future functional studies using gene expression validation, overexpression, silencing, or genome editing will be required to confirm the causal roles of the identified hub and neighbouring genes in grapevine salt tolerance.

## Data Availability

The RNA-Seq raw data for the ‘SO4’ rootstock are publicly available in the NCBI Sequence Read Archive (SRA) under accession number PRJNA1302591. The raw data supporting the findings for the ‘Bedia’ rootstock are available from the corresponding author upon reasonable request. All data generated or analyzed during this study are included in the source data files and Supplementary files.

## Supplementary Materials

The following supporting information can be downloaded at: https://www.mdpi.com/article/10.3390/ijms27146479/s1.

## References

[1] Hakeem A., Li S., Iqbal S., Elatafi E., Aziz R.B., Mauligen E., Liu S., Chen X., Zhang R., Shangguan L. (2025). Effects of salt stress on growth, physio-biochemical traits, and tolerance mechanism of grapevine rootstocks. Euphytica.

[2] FAO (2021). World Map of Salt-Affected Soils Launched at Virtual Conference. https://www.fao.org/newsroom/detail/salt-affected-soils-map-symposium/en.

[3] FAO (2024). Global Status of Salt-Affected Soils—Main Report.

[4] Mwesige F.F. (2025). The extent and distribution of salt-affected soils in sub-Saharan Africa from 1970 to the present: A review of the current state of knowledge. Front. Soil Sci..

[5] Mustapha A., Hakeem A., Li S., Mustafa G., Elatafi E., Fang J., Zhou C. (2025). Grapevine Rootstocks and Salt Stress Tolerance: Mechanisms, Omics Insights, and Implications for Sustainable Viticulture. Int. J. Plant Biol..

[6] Hasanuzzaman M., Fujita M. (2022). Plant responses and tolerance to salt stress: Physiological and molecular interventions. Int. J. Mol. Sci..

[7] Ullah A., Bano A., Khan N. (2021). Climate change and salinity effects on crops and chemical communication between plants and plant growth-promoting microorganisms under stress. Front. Sustain. Food Syst..

[8] Iqbal S., Elatafi E., Tariq K., Liu W., Elhendawy B., Shaonan L., Wang Y., Li W., Yu H., Fang J. (2026). Methyl Jasmonate Enhances Physiological and Biochemical Indicators and Alleviates Drought Stress in Grapevines Through the Activation of Genes *VvNCED1* and *VvABF1*. J. Plant Growth Regul..

[9] Shrivastava P., Kumar R. (2015). Soil salinity: A serious environmental issue and plant growth promoting bacteria as one of the tools for its alleviation. Saudi J. Biol. Sci..

[10] Atta K., Mondal S., Gorai S., Singh A.P., Kumari A., Ghosh T., Roy A., Hembram S., Gaikwad D.J., Mondal S. (2023). Impacts of salinity stress on crop plants: Improving salt tolerance through genetic and molecular dissection. Front. Plant Sci..

[11] Ji X., Tang J., Zhang J. (2022). Effects of salt stress on the morphology, growth and physiological parameters of *Juglans microcarpa* L. Seedlings. Plants.

[12] Atiyah F., Hamad R., Hammad M. (2025). Impact of Salt Stress on Pea (Pisum sativum) Physiological Features in Lab Settings. Razi Med. J..

[13] Han Y., Li X. (2024). Current progress in research focused on salt tolerance in *Vitis vinifera* L.. Front. Plant Sci..

[14] Elatafi E., Elhendawy B., Iqbal S., Ali S., Elshahat A., Hakeem A., Shaonan L., Ibrahim A., Shangguan L., Fang J. (2025). Physicochemical, Metabolite, Osmolyte Synthesis, and Enzymatic Alterations of Grapevine Rootstocks in Response to Cadmium Stress. J. Soil Sci. Plant Nutr..

[15] Elatafi E., Elshahat A., Xue Y., Shaonan L., Suwen L., Tianyu D., Fang J. (2023). Effects of different storage temperatures and methyl jasmonate on grape quality and antioxidant activity. Horticulturae.

[16] Yağcı A., Daler S., Kaya O. (2024). An Innovative Approach: Alleviating Cadmium Toxicity in Grapevine Seedlings Using Smoke Solution Derived from the Burning of Vineyard Pruning Waste. Physiol. Plant..

[17] Yu H., Elatafi E., Liu W., Zhang R., Elhendawy B., Xie S., Cao X., Bai X., Huang Q., Jiang C. (2026). Transcriptome Profiling of Powdery Mildew-Stressed ‘Yeniang No. 2’ Grapevine Reveals Differential Expression, Alternative Splicing, and the Identification of 1232 Annotated Novel Genes. Metabolites.

[18] FAOSTAT (2023). Food and Agriculture Organization of the United Nations. https://www.fao.org/faostat/en/#data/QCL.

[19] Wu J., Liu L., Xu G., Jianfu J., Lian W., Zhou H., Nan L., Ren H. (2021). Grape planting situation and regional spatial analysis in Xinjiang, China. IOP Conf. Ser. Earth Environ. Sci..

[20] Iqbal S., Elatafi E., Shaonan L., Ali S., Hakeem A., Badar Aziz R., Mauligen E., Tariq K., Elhendawy B., Shangguan L. (2025). Drought Stress and the Modulation of Physiochemical Parameters and Antioxidant Enzymes in Grapevine Rootstocks: Insights into the Protective Role of Methyl Jasmonate. Horticulturae.

[21] Hakeem A., Li S., Nasiru M.M., Mustafa G., Elatafi E., Shangguan L., Fang J. (2025). Methyl Jasmonate Acts as a Crucial Player in Abiotic Stress Responses in Grape. Stresses.

[22] Elatafi E., Elhendawy B., Elshahat A., Iqbal S., Yauha R., Xuan X., Li W., Li F., Fan S., Hakeem A. (2025). A Comprehensive Analysis of Cadmium Contamination in Viticulture: From Soil and Grape to Ecological Risks and Remediation. J. Soil Sci. Plant Nutr..

[23] Yan H., Elatafi E., Iqbal S., Chen Y., Yang G., Zhang Y., Li X., Li B., Liu K., Wu Y. (2026). Transcriptomic analysis reveals a coordinated stress response and metabolic reprogramming in ‘Muscat Hamburg’ grape berries subjected to partial root-zone irrigation. Sci. Hortic..

[24] Xiao F., Zhou H. (2023). Plant salt response: Perception, signaling, and tolerance. Front. Plant Sci..

[25] Van Zelm E., Zhang Y., Testerink C.J. (2020). Salt tolerance mechanisms of plants. Annu. Rev. Plant Biol..

[26] Corso M., Bonghi C. (2014). Grapevine rootstock effects on abiotic stress tolerance. Horizon.

[27] Corso M., Vannozzi A., Ziliotto F., Zouine M., Maza E., Nicolato T., Vitulo N., Meggio F., Valle G., Bouzayen M. (2016). Grapevine rootstocks differentially affect the rate of ripening and modulate auxin-related genes in cabernet sauvignon berries. Front. Plant Sci..

[28] Lupo Y., Schlisser A., Dong S., Rachmilevitch S., Fait A., Lazarovitch N. (2022). Root system response to salt stress in grapevines (*Vitis* spp.): A link between root structure and salt exclusion. Plant Sci..

[29] Zhao F., Zheng T., Liu Z., Fu W., Fang J. (2022). Transcriptomic analysis elaborates the resistance mechanism of grapevine rootstocks against salt stress. Plants.

[30] Troncoso de Arce A., Matte C., Cantos M., Lavee S. (2015). Evaluation of salt tolerance of in vitro-grown grapevine rootstock varieties. Vitis.

[31] Bianchi D., Caramanico L., Grossi D., Brancadoro L., Lorenzis G.D. (2020). How do novel M-rootstock (*Vitis* spp.) genotypes cope with drought?. Plants.

[32] Chen Y., Fei Y., Howell K., Chen D., Clingeleffer P., Zhang P. (2024). Rootstocks for Grapevines Now and into the Future: Selection of Rootstocks Based on Drought Tolerance, Soil Nutrient Availability, and Soil pH. Aust. J. Grape Wine Res..

[33] Zhao B., Liu Z., Zhu C., Zhang Z., Shi W., Lu Q., Sun J. (2023). Saline–alkaline stress resistance of cabernet sauvignon grapes grafted on different rootstocks and rootstock combinations. Plants.

[34] Hussain Q., Asim M., Zhang R., Khan R., Farooq S., Wu J. (2021). Transcription factors interact with ABA through gene expression and signaling pathways to mitigate drought and salinity stress. Biomolecules.

[35] Li S., Khoso M.A., Xu H., Zhang C., Liu Z., Wagan S., Dinislam K., Liu L. (2024). WRKY transcription factors (TFs) as key regulators of plant resilience to environmental stresses: Current perspective. Agronomy.

[36] Lu X., Chen G., Ma L., Zhang C., Yan H., Bao J., Nai G., Wang W., Chen B., Ma S. (2023). Integrated transcriptome and metabolome analysis reveals antioxidant machinery in grapevine exposed to salt and alkali stress. Physiol. Plant..

[37] Alshiekheid M.A., Dwiningsih Y., Alkahtani J. (2023). Analysis of morphological, physiological, and biochemical traits of salt stress tolerance in Asian rice cultivars at different stages. Stresses.

[38] Soltabayeva A., Ongaltay A., Omondi J.O., Srivastava S. (2021). Morphological, physiological and molecular markers for salt-stressed plants. Plants.

[39] Mi J., Ren X., Shi J., Wang F., Wang Q., Pang H., Kang L., Wang C. (2024). An insight into the different responses to salt stress in growth characteristics of two legume species during seedling growth. Front. Plant Sci..

[40] Birhanie Z.M., Yang D., Luan M., Xiao A., Liu L., Zhang C., Biswas A., Dey S., Deng Y., Li D. (2022). Salt stress induces changes in physiological characteristics, bioactive constituents, and antioxidants in kenaf (*Hibiscus cannabinus* L.). Antioxidants.

[41] Kumar S., Li G., Yang J., Huang X., Ji Q., Liu Z., Ke W., Hou H. (2021). Effect of salt stress on growth, physiological parameters, and ionic concentration of water dropwort (*Oenanthe javanica*) cultivars. Front. Plant Sci..

[42] Wang M., Gong S., Fu L., Hu G., Li G., Hu S., Yang J. (2022). The involvement of antioxidant enzyme system, nitrogen metabolism and osmoregulatory substances in alleviating salt stress in inbred maize lines and hormone regulation mechanisms. Plants.

[43] Yağcı A. (2025). Shikonin-induced secondary metabolite modulation in grape berries (*Vitis vinifera* L. cv.‘Öküzgözü’) under salinity stress. BMC Plant Biol..

[44] Malik A.A., Li W.G., Lou L.N., Weng J.H., Chen J.F. (2010). Biochemical/physiological characterization and evaluation of in vitro salt tolerance in cucumber. Afr. J. Biotechnol..

[45] Sarker U., Oba S. (2020). The response of salinity stress-induced A. tricolor to growth, anatomy, physiology, non-enzymatic and enzymatic antioxidants. Front. Plant Sci..

[46] Wang D., Gao Y., Sun S., Lu X., Li Q., Li L., Wang K., Liu J. (2022). Effects of salt stress on the antioxidant activity and malondialdehyde, solution protein, proline, and chlorophyll contents of three Malus species. Life.

[47] Mozafari A.-A., Ghadakchi asl A., Ghaderi N. (2018). Grape response to salinity stress and role of iron nanoparticle and potassium silicate to mitigate salt induced damage under in vitro conditions. Physiol. Mol. Biol. Plants.

[48] Shaikhaldein H.O., Al-Qurainy F., Nadeem M., Khan S., Tarroum M., Salih A.M., Al-Hashimi A. (2024). Biosynthesis of copper nanoparticles using Solenostemma argel and their effect on enhancing salt tolerance in barley plants. Sci. Rep..

[49] Barakat A.A., Hussein B.A., Awad N., Soliman M.H. (2019). Evaluation of the two rootstocks (SO_4_ and Freedom) for the salt stress in vitro conditions. Plant Arch..

[50] Habib A., Ben Maachia S., Namsi A., Harbi Ben Slimane M., Jeandet P., Aziz A. (2023). Evaluation of Salt Stress-Induced Changes in Polyamine, Amino Acid, and Phytoalexin Profiles in Mature Fruits of Grapevine Cultivars Grown in Tunisian Oases. Plants.

[51] Hossain M.A., Bhattacharjee S., Armin S.-M., Qian P., Xin W., Li H.-Y., Burritt D.J., Fujita M., Tran L.-S.P. (2015). Hydrogen peroxide priming modulates abiotic oxidative stress tolerance: Insights from ROS detoxification and scavenging. Front. Plant Sci..

[52] Khedia J., Agarwal P., Agarwal P.K. (2019). Deciphering hydrogen peroxide-induced signalling towards stress tolerance in plants. 3 Biotech.

[53] Goncharuk E.A., Zubova M.Y., Nechaeva T.L., Kazantseva V.V., Gulevich A.A., Baranova E.N., Lapshin P.V., Katanskaya V.M., Aksenova M.A., Zagoskina N.V. (2022). Effects of Hydrogen Peroxide on In Vitro Cultures of Tea (*Camellia sinensis* L.) Grown in the Dark and in the Light: Morphology, Content of Malondialdehyde, and Accumulation of Various Polyphenols. Molecules.

[54] Maggiolini F.A.M., Santamaria M., Bergamini C., Marsico A.D., Basile T., Forleo L.R., Prencipe A., Perniola R., D’Amico M., Cardone M.F. (2026). Scion-Rootstock interactions shape early transcriptional responses to controlled water deficit in newly selected table grape genotypes. BMC Plant Biol..

[55] Rao M.J., Duan M., Zhou C., Jiao J., Cheng P., Yang L., Wei W., Shen Q., Ji P., Yang Y. (2025). Antioxidant Defense System in Plants: Reactive Oxygen Species Production, Signaling, and Scavenging During Abiotic Stress-Induced Oxidative Damage. Horticulturae.

[56] Alharbi K., Al-Osaimi A.A., Alghamdi B.A. (2022). Sodium chloride (NaCl)-induced physiological alteration and oxidative stress generation in *Pisum sativum* (L.): A toxicity assessment. ACS Omega.

[57] Prinsi B., Failla O., Scienza A., Espen L. (2020). Root proteomic analysis of two grapevine rootstock genotypes showing different susceptibility to salt stress. Int. J. Mol. Sci..

[58] Fracasso A., Trindade L.M., Amaducci S. (2016). Drought stress tolerance strategies revealed by RNA-Seq in two sorghum genotypes with contrasting WUE. BMC Plant Biol..

[59] Yang Z., Lu R., Dai Z., Yan A., Tang Q., Cheng C., Xu Y., Yang W., Su J. (2017). Salt-stress response mechanisms using de novo transcriptome sequencing of salt-tolerant and sensitive *Corchorus* spp. genotypes. Genes.

[60] Muthusamy M., Uma S., Backiyarani S., Saraswathi M.S., Chandrasekar A. (2016). Transcriptomic changes of drought-tolerant and sensitive banana cultivars exposed to drought stress. Front. Plant Sci..

[61] Dalal M., Inupakutika M. (2014). Transcriptional regulation of ABA core signaling component genes in sorghum (*Sorghum bicolor* L. Moench). Mol. Breed..

[62] Ojosnegros S., Alvarez J.M., Grossmann J., Gagliardini V., Quintanilla L.G., Grossniklaus U., Fernández H. (2023). Proteome and interactome linked to metabolism, genetic information processing, and abiotic stress in gametophytes of two woodferns. Int. J. Mol. Sci..

[63] Lin Y., Liu S., Fang X., Ren Y., You Z., Xia J., Hakeem A., Yang Y., Wang L., Fang J. (2023). The physiology of drought stress in two grapevine cultivars: Photosynthesis, antioxidant system, and osmotic regulation responses. Physiol. Plant..

[64] Chen J., Hu Y., Zhao T., Huang C., Chen J., He L., Dai F., Chen S., Wang L., Jin S. (2024). Comparative transcriptomic analysis provides insights into the genetic networks regulating oil differential production in oil crops. BMC Biol..

[65] Zhang Y., Dong W., Ma H., Zhao C., Ma F., Wang Y., Zheng X., Jin M. (2024). Comparative transcriptome and coexpression network analysis revealed the regulatory mechanism of *Astragalus cicer* L. in response to salt stress. BMC Plant Biol..

[66] Cortes A. (2024). Abiotic Stress Tolerance Boosted by Genetic Diversity in Plants. Int. J. Mol. Sci..

[67] Zeng Z., Zhang S., Li W., Chen B., Li W. (2022). Gene-coexpression network analysis identifies specific modules and hub genes related to cold stress in rice. BMC Genom..

[68] de Abreu C.E., Araújo Gdos S., Monteiro-Moreira A.C., Costa J.H., Leite Hde B., Moreno F.B., Prisco J.T., Gomes-Filho E. (2014). Proteomic analysis of salt stress and recovery in leaves of Vigna unguiculata cultivars differing in salt tolerance. Plant Cell Rep..

[69] Chen M., Han G., Dietrich C.R., Dunn T.M., Cahoon E.B. (2006). The essential nature of sphingolipids in plants as revealed by the functional identification and characterization of the Arabidopsis LCB1 subunit of serine palmitoyltransferase. Plant Cell.

[70] Kimberlin A.N., Majumder S., Han G., Chen M., Cahoon R.E., Stone J.M., Dunn T.M., Cahoon E.B. (2013). Arabidopsis 56-amino acid serine palmitoyltransferase-interacting proteins stimulate sphingolipid synthesis, are essential, and affect mycotoxin sensitivity. Plant Cell.

[71] Liu X., Zhu Y., Zhai H., Cai H., Ji W., Luo X., Li J., Bai X. (2012). AtPP2CG1, a protein phosphatase 2C, positively regulates salt tolerance of Arabidopsis in abscisic acid-dependent manner. Biochem. Biophys. Res. Commun..

[72] Chen X., Chen G., Guo S., Wang Y., Sun J. (2023). SlSAMS1 enhances salt tolerance through regulation DNA methylation of SlGI in tomato. Plant Sci..

[73] Yuan F., Yang H., Xue Y., Kong D., Ye R., Li C., Zhang J., Theprungsirikul L., Shrift T., Krichilsky B. (2014). OSCA1 mediates osmotic-stress-evoked Ca2+ increases vital for osmosensing in Arabidopsis. Nature.

[74] Li P., Cao W., Fang H., Xu S., Yin S., Zhang Y., Lin D., Wang J., Chen Y., Xu C. (2017). Transcriptomic profiling of the maize (*Zea mays* L.) leaf response to abiotic stresses at the seedling stage. Front. Plant Sci..

[75] Song X., Duan Y.-Y., Tan F.-Q., Ren J., Cao H.-X., Xie K.-D., Wu X.-M., Guo W.-W. (2023). Comparative transcriptome analysis of salt tolerance of roots in diploid and autotetraploid citrus rootstock (*C. junos* cv. Ziyang xiangcheng) and identification of salt tolerance-related genes. Sci. Hortic..

[76] Joshi R., Wani S.H., Singh B., Bohra A., Dar Z.A., Lone A.A., Pareek A., Singla-Pareek S.L. (2016). Transcription factors and plants response to drought stress: Current understanding and future directions. Front. Plant Sci..

[77] Iqbal S., Elatafi E., Tariq K., Ali S., Hakeem A., Shaonan L., Aziz R.B., Mauligen E.Q., Fang J. (2025). Drought Stress in Viticulture: An Update Review of the Effects, Mechanisms, Tolerance Strategies, and Mitigation Approaches. J. Soil Sci. Plant Nutr..

[78] Elatafi E., Iqbal S., Elhendawy B., Marey S.A., Ali S., Hakeem A., Baiome B.A., Elshahat A., Ibrahim A., Fang J. (2026). Grapevine rootstocks are a promising strategy for phytostabilization in soils contaminated with cadmium. Sci. Rep..

[79] Saeed M., Zhao H., Chen Z., Ju P., Wang G., Zhou C., Jia H., Zhu C., Jia H., Jiao Y. (2024). Wax bayberry is a suitable rootstock for Chinese red bayberry cultivated in saline-alkali soil. Sci. Hortic..

[80] Hoagland D., Arnon D. (1950). The water culture method forgrowing plants without soil. Calif. Agric. Exp. Stn. Circ..

[81] Shafqat W., Jaskani M.J., Maqbool R., Khan A.S., Ali Z. (2019). Evaluation of citrus rootstocks against drought, heat and their combined stress based on growth and photosynthetic pigments. Int. J. Agric. Biol..

[82] Peng Y., Jin T., Yang R., Chen R., Wang J. (2022). Distribution of mineral elements in the soil and in tea plants (*Camellia sinensis*). J. Elem..

[83] Bian S., Jiang Y. (2009). Reactive oxygen species, antioxidant enzyme activities and gene expression patterns in leaves and roots of Kentucky bluegrass in response to drought stress and recovery. Sci. Hortic..

[84] Giannopolitis C., Ries S. (1977). Superoxide Dismutases: I. Occurrence in Higher Plants. Plant Physiol..

[85] Guo T.-R., Zhang G.P., Zhou M., Wu F., Chen J. (2004). Effects of aluminum and cadmium toxicity on growth and antioxidant enzyme activities of two barley genotypes with different Al resistance. Plant Soil.

[86] Durner J., Klessig D.F. (1996). Salicylic acid is a modulator of tobacco and mammalian catalases. J. Biol. Chem..

[87] Nakano Y., Asada K. (1981). Hydrogen peroxide is scavenged by ascorbate-specific peroxidase in spinach chloroplasts. Plant Cell Physiol..

[88] Lowry O.H., Rosebrough N.J., Farr A.L., Randall R.J. (1951). Protein measurement with the Folin phenol reagent. J. Biol. Chem..

[89] Heath R.L., Packer L. (1968). Photoperoxidation in isolated chloroplasts: I. Kinetics and stoichiometry of fatty acid peroxidation. Arch. Biochem. Biophys..

[90] Rhee S.G., Chang T.-S., Jeong W., Kang D. (2010). Methods for detection and measurement of hydrogen peroxide inside and outside of cells. Mol. Cells.

[91] Abdelrady W.A., Ma Z., Elshawy E.E., Wang L., Askri S.M.H., Ibrahim Z., Dennis E., Kanwal F., Zeng F., Shamsi I.H. (2024). Physiological and biochemical mechanisms of salt tolerance in barley under salinity stress. Plant Stress.

[92] Dong T., Zhang P., Hakeem A., Liu Z., Su L., Ren Y., Pei D., Xuan X., Li S., Fang J. (2023). Integrated transcriptome and metabolome analysis reveals the physiological and molecular mechanisms of grape seedlings in response to red, green, blue, and white LED light qualities. Environ. Exp. Bot..

[93] Wang L., Feng Z., Wang X., Wang X., Zhang X. (2010). DEGseq: An R package for identifying differentially expressed genes from RNA-seq data. Bioinformatics.

[94] Kanehisa M., Goto S. (2000). KEGG: Kyoto encyclopedia of genes and genomes. Nucleic Acids Res..

[95] Langfelder P., Wgcna S. (2008). An R package for weighted correlation network analysis. BMC Bioinform..

[96] Dong T., Hao T., Hakeem A., Ren Y., Fang J. (2024). Synergistic variation in abscisic acid and brassinolide treatment signaling component alleviates fruit quality of ‘Shine Muscat’ grape during cold storage. Food Chem..

[97] Livak K.J., Schmittgen T.D. (2001). Analysis of relative gene expression data using real-time quantitative PCR and the 2−ΔΔCT method. Methods.

