# Supplementary material for: Transcriptomic and Physiological Profiling Elucidates Differential Salt Stress Responses in Tolerant ‘SO4’ and Sensitive ‘Beida’ Grapevine Rootstocks
Source: Int J Mol Sci. 2026 Jul 21;27(14):6479. doi: 10.3390/ijms27146479 (PMC13411216; doi:10.3390/ijms27146479)
Supplement: Supplementary file 1 [file ijms-27-06479-s001.zip › Supplementary Figures.pdf]

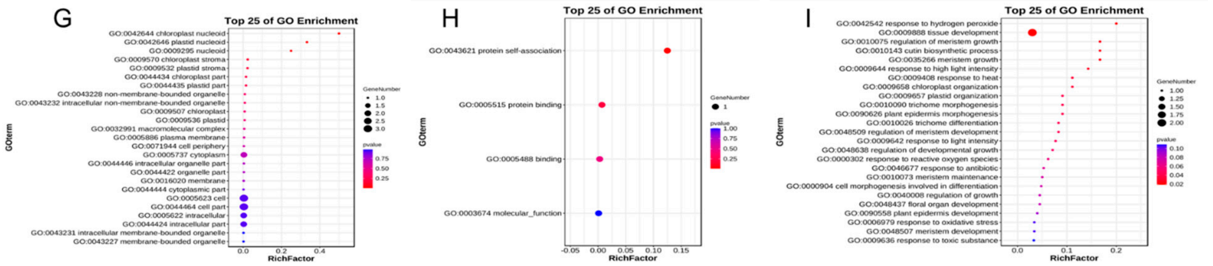

Figure S1. Illustrates the key pathways in the gene ontology enrichment visualization between both grapevine rootstocks under stress treatments. The letter shows A (0d_CK), B (0d_NaCl), C (6d_CK), D (6d_NaCl), E (12d_CK), F (12d_NaCl), G (0d_CK), H (0d_NaCl), I (6d_CK), J (6d_NaCl), K (12d_CK), L (12d_NaCl).

(A)

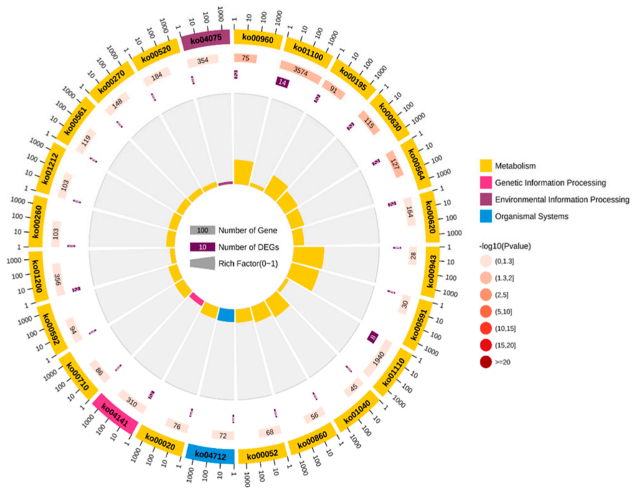

(B)

Pathway ID	Description
ko00960	Tropane, piperidine and pyridine alkaloid biosynthesis
ko00960	Metabolic pathways
ko01100	Photosynthesis
ko00195	Glyoxylate and dicarboxylate metabolism
ko00630	Glycerophospholipid metabolism
ko00564	Pyruvate metabolism
ko00620	Isoflavonoid biosynthesis
ko00943	Linoleic acid metabolism
ko00943	Biosynthesis of secondary metabolites
ko00591	Biosynthesis of unsaturated fatty acids
ko00052	Porphyrin metabolism
ko00860	Galactose metabolism
ko04712	Circadian rhythm - plant
ko00020	Citrate cycle (TCA cycle)
ko04141	Protein processing in endoplasmic reticulum
ko00710	Carbon fixation in photosynthetic organisms
ko00592	alpha-Linolenic acid metabolism
ko01200	Carbon metabolism
ko00260	Glycine, serine and threonine metabolism
ko01212	Fatty acid metabolism
ko00561	Glycerolipid metabolism
ko00270	Cysteine and methionine metabolism
ko00520	Amino sugar and nucleotide sugar metabolism
ko04075	Plant hormone signal transduction

Figure S2. Circle chart and enrichment analysis displaying the findings about 24 KO terms between 72 DEGs in both rootstock cultivars under salt stress. From outdoor to indoor, the first circle illustrates the distribution of enriched categories, with the coordinate axis displaying the number of genes outside the circle. The second circle represents the number of genes and p-values in the background genes. And, the third circle illustrates several differentially expressed genes (A). Enrichment analysis of 72 DEGs in both grapevine rootstocks was mainly attached to four metabolic pathways including metabolism, organismal system, genetic information processing, and environmental information processing (B). Note: specific values are shown below.

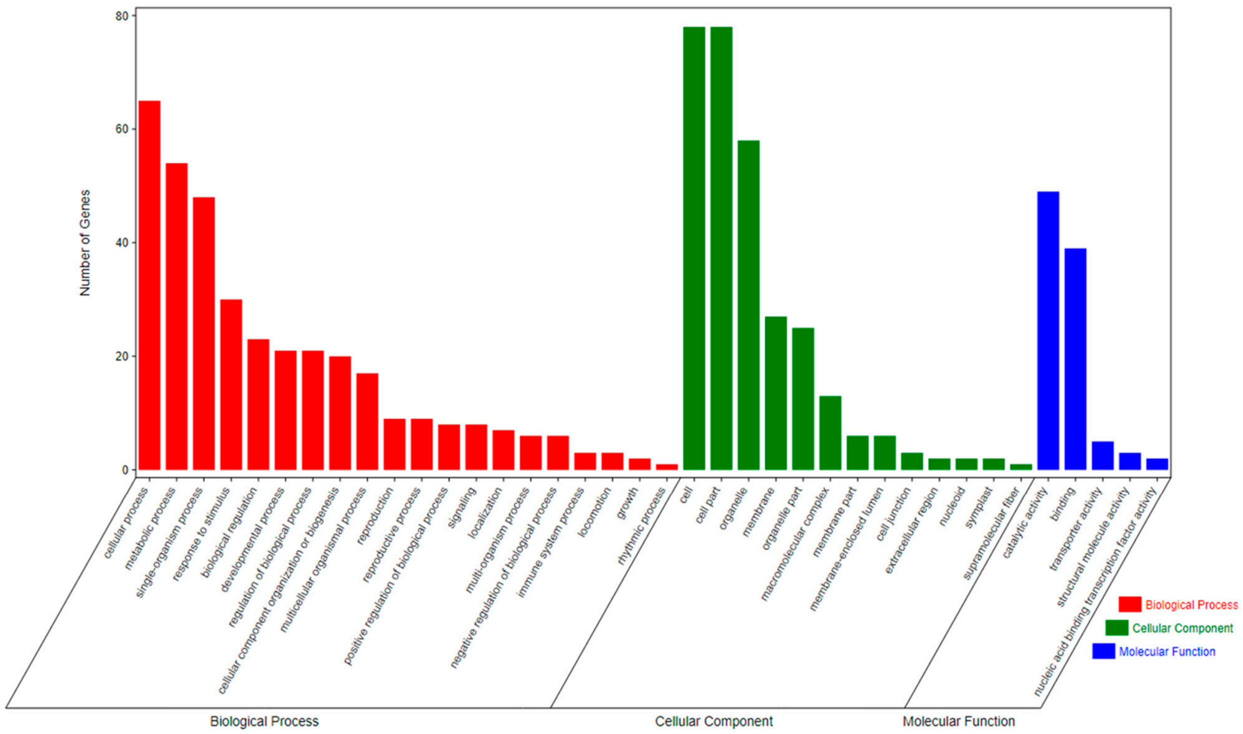

Figure S3. Represents the biological process, cellular components, and molecular function based on Profile 0 and 5, respectively.

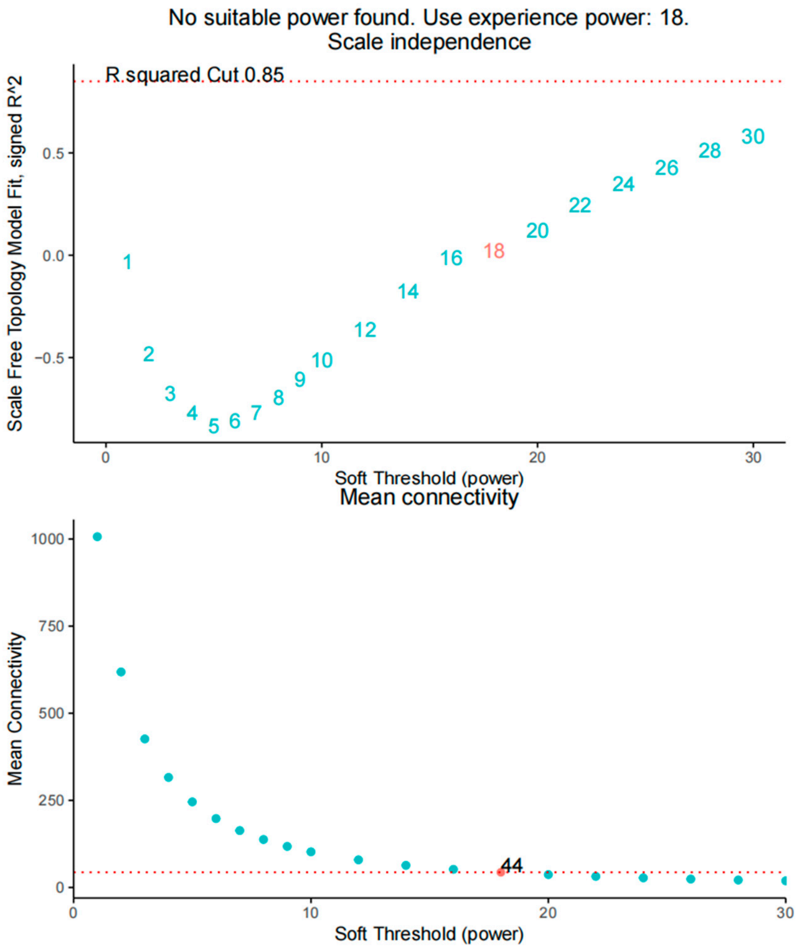

Figure S4. Interpretation of coexpression and WGCNA analysis of salt stress responses of DEGs in 'SO4' and 'Beida' grapevine rootstocks. A soft power curve, the ordinate on the left side shows the construction coefficient, while the right side shows the construction of the average connectivity between DEGs.

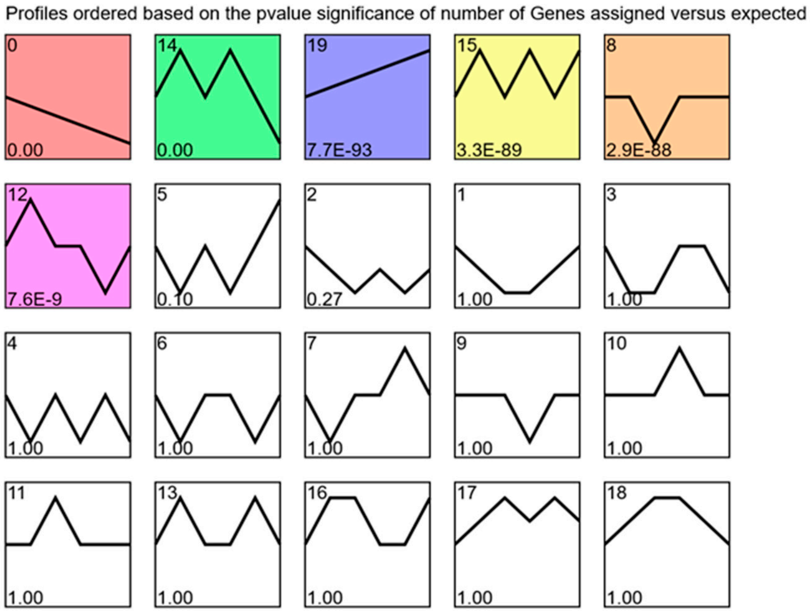

Figure S5. Trend analysis of gene expression profiles in grapevine grafted onto 'SO4' and 'Beida' rootstocks. Each box represents a specific expression profile. Profiles are ordered based on the statistical significance (*p*-value) of the number of genes assigned to each profile compared to the expected number. The x-axis represents the time points 0, 6, and 12 days, and the y-axis represents the relative change in gene expression. Colored profiles (Profiles 0, 14, 19, 15, 8, and 12) indicate statistically significant clusters ($p < 0.05$). The number at the top left of each box is the profile ID, and the number at the bottom left is the associated *p*-value.

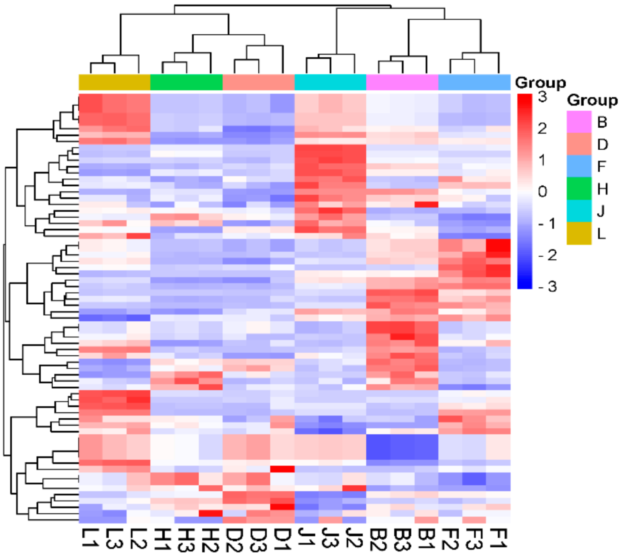

Figure S6. Heatmap of differentially expressed transcription factors WRKY in 'SO4', and 'Beida' grapevine rootstock under salt stress (NaCl; 100 mmol L^{-1}). Represents the enriched TFs between 0, 6, and 12 d of stress duration adjacent time (B1, B2, B3: 0d), (D1, D2, D3: 6d), and (F1, F2, F3: 12 d) in 'SO4'. Similarly (H1, H2, H3: 0d), (J1, J2, J3: 6d), and (L1, L2, L3: 12 d) in 'Beida' grapevine rootstocks.
